# Benzene-fused bis(acenaphthoBODIPY)s, stable near-infrared-selective dyes†

**DOI:** 10.1039/c8ra01694a

**Published:** 2018-04-16

**Authors:** Hidemitsu Uno, Takayuki Honda, Manami Kitatsuka, Shogo Hiraoka, Shigeki Mori, Masayoshi Takase, Tetsuo Okujima, Takahiro Nakae

**Affiliations:** Department of Chemistry and Biology, Graduate School of Science and Engineering, Ehime University 2-5 Bunkyo-cho Matsuyama 790-8577 Japan uno@ehime-u.ac.jp; Division of Material Science, Advanced Research Support Center, Ehime University 2-5 Bunkyo-cho Matsuyama 790-8577 Japan; Institute of Advanced Energy, Kyoto University Gokasyo Uji 711-0011 Japan

## Abstract

Benzene-fused bis(acenaphthoBODIPY)s prepared by retro-Diels–Alder reaction of bicyclo[2.2.2]octadiene-fused precursors showed strong absorption bands in the near-infrared region and very weak absorptions in the visible region.

## Graphical abstract

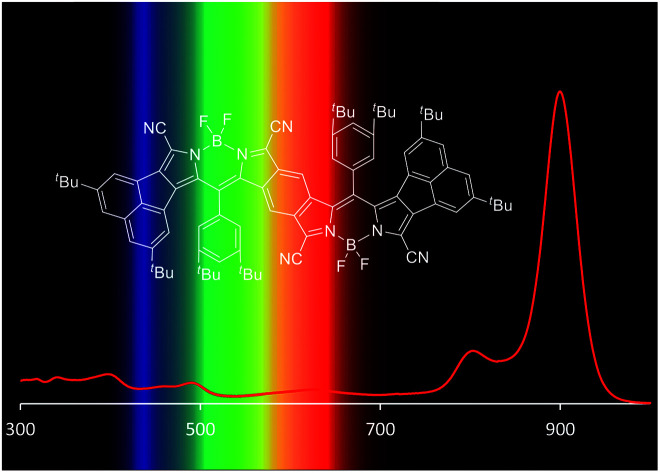

## Introduction

**Fig. 1:**
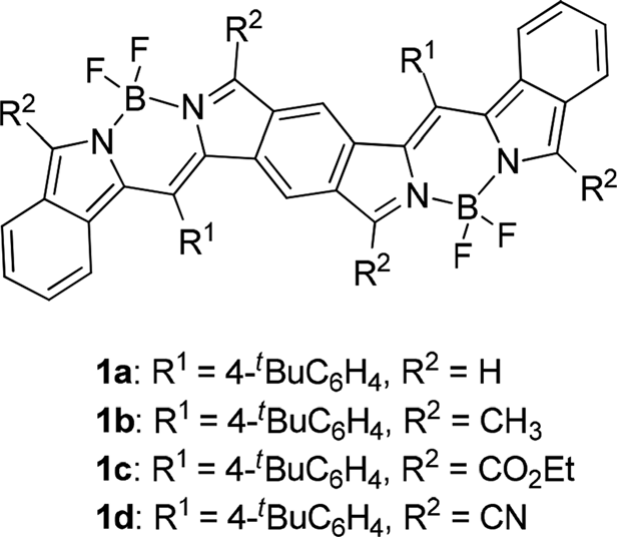
Benzene-fused bis(benzoBODIPY)s with NIR absorptions.

Biological and clinical applications of near-infrared (NIR) dyes is an area of great interest because the development of laser-generating techniques enables us to use light of a certain wavelength more easily than before. In the medical fields, NIR light in the so-called optical window region plays a key role in photodynamic therapy for cancer treatment^1^ and imaging of mammalian living cells in deep tissue^2^ due to its permeability. In industry, organic NIR dyes are a key material for improving the efficiency of organic solar cells;^3^ fair mounts of solar energy reach the earth's surface as NIR light. Inorganic NIR dyes are used as cutoff filters for detectors and as ink for NIR-reading machines.^4^ NIR dyes that have no or little absorption in the visible region can be used as invisible NIR ink for humans, whereas panchromatic NIR dyes are ideal for solar-cell application. Many kinds of organic compounds that are used as NIR dyes usually have large π chromophores such as linear cyanine,^5^ cyclic oligopyrroles including porphyrin and phthalocyanine,^6^ squaraine,^7^ rhodamine^8^ and boron dipyrromethene (BODIPY);^9^ however, absorption maxima of their pristine chromophores, except for cyclo[*n*]pyrroles,^10^ do not reach the NIR region. Expansion of the core chromophores or efficient introduction of substituents is therefore essential for dyes with NIR absorption. We have approached this subject by fusion and expansion of chromophores. We reported that π-expanded porphyrins,^11^ π-expanded BODIPYs,^12^ π-fused oligoporphyrins^13^ and benzene-fused bisBODIPYs^14^ have strong absorption maxima in the far-red-to-NIR region. Especially in the case of benzene-fused bis(benzoBODIPY)s 1 (Fig. 1), only the absorption maxima with the lowest energy occurred in the NIR region (775 to 903 nm), depending on the substituents. Both panchromatic BODIPYs for solar-cell application^15^ and NIR-selective BODIPYs for bio-imaging application^9^ have attracted much attention. Our fusion method for the BODIPY chromophore in the proper direction was proven to be effective for the elongation of absorption maxima into the NIR region with keeping transparency in the visible region. Similar to the π-expanded porphyrins^11^ and BODIPYs,^12^ however, bis(benzoBODIPY)s 1a and 1b without electron-withdrawing groups was proven to be unstable. Although introduction of electron-withdrawing groups such as ethoxycarbonyl and cyano groups stabilized the benzene-fused bis(benzoBODIPY) chromophore, the absorption maxima also tended to shift bathochromically. In order to tune absorption maxima of the NIR dyes, stable NIR π systems based on the BODIPY chromophore are necessary. Lash and coworkers reported that the absorption maxima of porphyrin Q bands are remarkably bathochromically shifted in the NIR region by fusion of acenaphtho moieties to β positions of porphyrins.^16^ We have also reported the effective elongation of absorption maxima in the case of BODIPYs^17^ and cyclo[*n*]pyrroles.^18^ This acenaphtho-fusion method was successfully applied to the preparation of bisBODIPYs with stable NIR chromophores.

## Results and discussion

**Scheme 1:**
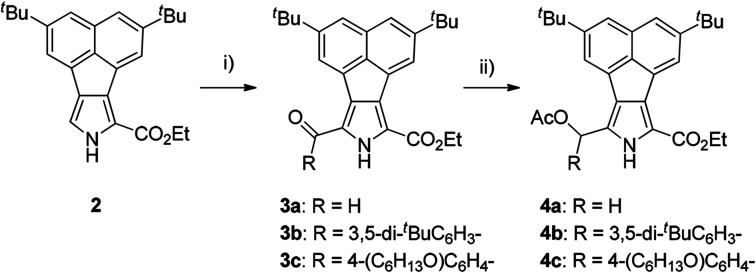
Reagents, conditions and yields: (i) for 3a: DMF, POCl_3_, rt, 1.5 h; 98%; 3b: 3,5-di-*tert*-butylbenzoic acid, trifluoroacetic anhydride, trifluoroacetic acid, rt, 3 d; 80%; 3c: 4-hexyloxybenzoic acid, TFAA, TFA, rt, 3 d; 96%. (ii) NaBH_4_, THF/EtOH (2/1), 0 °C, 2 h; Ac_2_O, DMAP (cat.), CH_2_Cl_2_, rt, 30 min; 4a: 92%, 4b: 69%, 4c: 65%.

Our strategy for the preparation of acenaphtho-fused bisBODIPYs (bisANBODIPYs) was based on the retro-Diels–Alder protocol,^19^ which was successfully used in the preparation of highly flat, insoluble compounds. This protocol enabled us to treat the intermediary compounds in the synthetic scheme without worrying about their solubility, and it was quite successful when the final targeted compounds did not need to be characterized. In most cases, the solubility of the targeted compounds was required for their full characterization. In order to increase the solubility of the target acenaphtho derivatives in common organic solvents, two *tert*-butyl groups were introduced into acenaphthylene, and the resulting compound was converted to ethylacenaphtho[1,2-*c*]pyrrole-1-carboxylate 2.^18^ Formylation of 2 with the Vilsmeier reagent gave α-formylated compound 3a in 98% yield (Scheme 1). The formyl group of 3a was converted to an acetoxymethyl group by reduction with NaBH_4_ followed by acetylation with Ac_2_O and 4-dimethylaminopyridine (DMAP). Compound 4a was obtained in 92% yield in two steps.

**Scheme 2:**
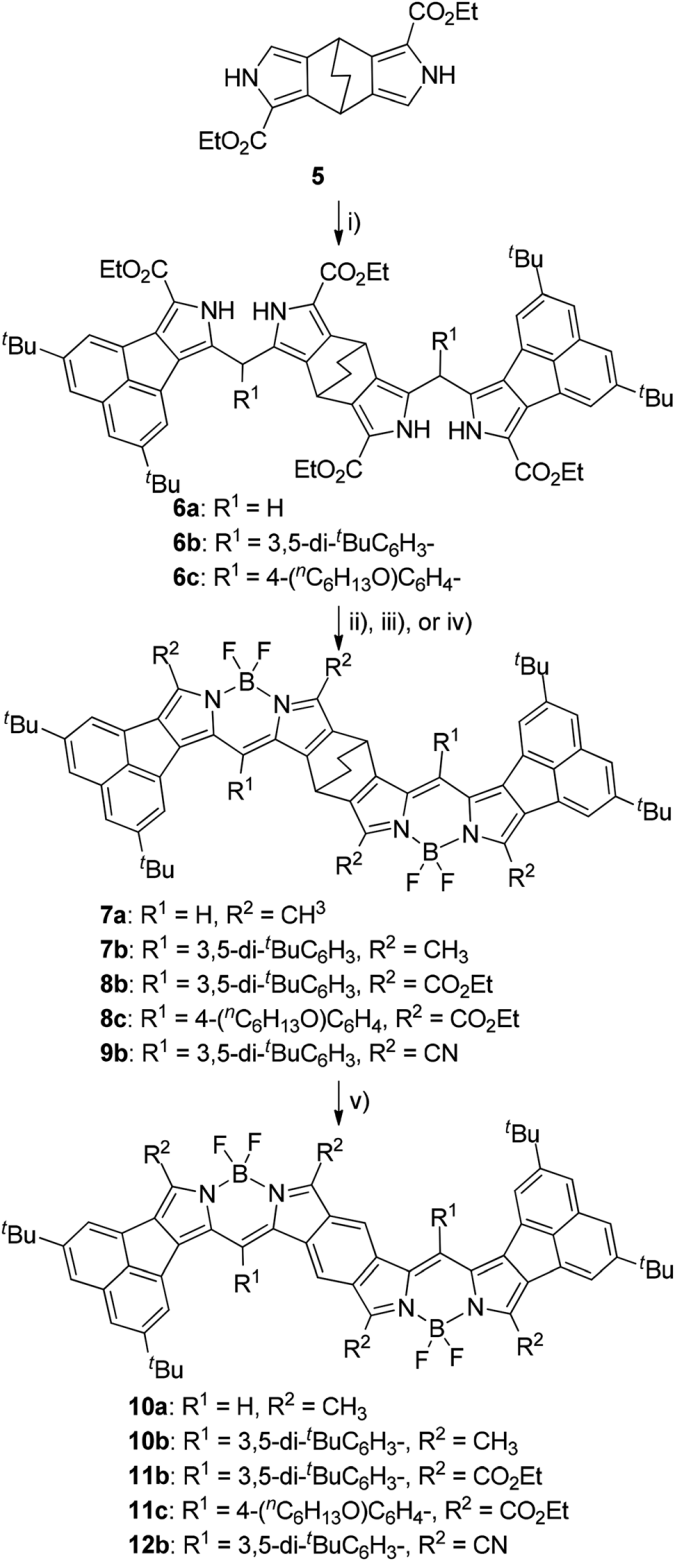
Reagents, conditions and yields: (i) 4, TsOH, AcOH, rt, 3 h; 6a: 63%; 6b: 58%; 6c: 75%; (ii) LiAlH_4_, THF, reflux, 3 h; DDQ, CH_2_Cl_2_, rt, 1 h; BF_3_·OEt_2_, (i-Pro)_2_EtN, 2 h; 7a: 27%; 7b: 35%; (iii) DDQ, CH_2_Cl_2_, rt, 1 h; BF_3_·OEt_2_, (i-Pro)_2_EtN, 2 h; 8b: 70%; 8c: 80%; (iv) NaOH, ethylene glycol, 170 °C, 3 h; CSI, DMF/CH_3_CN (2 : 1), −50 °C, 1.5 h, then rt, overnight; DDQ, CH_2_Cl_2_, rt, 1 h; BF_3_·OEt_2_, *N*,*N*-diisopropylethylamine, 2 h; 5%; 9b; (v) 200 °C, 2 h or 250 °C, 30 min; quant.

We have reported that the fusion geometry of two BODIPY chromophores is very important for the elongation of absorption maxima with the lowest energy: fusion of the chromophores in the anti manner was found to be more effective than the was *syn* manner.^14^ Therefore, we chose bicyclo[2.2.2]octadiene-fused (BCOD-fused) dipyrroles 5 (ref. 20) as the starting material. Double condensation of 4a with 5 under acidic conditions gave BCOD-fused bis(dipyrromethene)s 6a in 66% yield (Scheme 2). Four ester groups of 6a were transformed to four methyl groups by rigorous reduction with LiAlH_4_ in refluxing THF in 37% yield. The rather unstable tetramethyl derivative was then converted to BCOD-fused bis(boron acenaphthodipyrromethene) (BCOD-bisANBODIPY) 7a in 27% yield by treatment with 2,3-dichloro-5,6-dicyano-*p*-benzoquinone (DDQ), (i-Pr)_2_EtN and BF_3_·OEt_2_. The thermogravimetric experiment on precursor 7a showed that evolution of ethylene gas started from *ca.* 200 °C and ceased at 250 °C (temperature increase rate of 10 °C min^−1^). The starting reddish purple color of 7a turned black. Thus, the bulk thermal conversion of 7a was performed at 200 °C for 2 h *in vacuo*. The obtained material was poorly soluble and no purification was possible, although the ultraviolet-visible-NIR (UV-vis-NIR) spectrum and matrix-assisted laser-ionization time-of-flight (MALDI-TOF) MS of the material indicated the formation of benzene-fused bisANBODIPY (B-bisANBODIPY) 10a (Scheme 2).

We next examined the use of solubilizing substituents such as 3,5-di-*tert*-butylphenyl and 4-hexyloxyphenyl groups in order to characterize the B-bisANBODIPYs. Thus, acetoxy(aryl)methyl derivatives 4b and 4c were prepared (Scheme 1). Friedel–Crafts acylation of 2 by the mixed anhydrides of 3,5-di-*tert*-butylbenzoic acid and 4-hexyloxybenzoic acid with trifluoroacetic anhydride (TFAA) and trifluoroacetic acid (TFA) afforded α-acylated compounds 3b and 3c in respective yields of 80 and 96%.^21^ Reduction of the acyl groups of 3 with NaBH_4_ followed by acetylation with Ac_2_O gave α-acetoxymethyl derivatives 4b and 4c in 69% and 65% yields, respectively. Treatment of 4b with 5 under acidic conditions afforded a diastereomeric mixture of BCOD-fused bis(dipyrromethane) 6b in 58% yield (Scheme 2). Bis(dipyrromethane) 6b was transformed into three types of BCOD-bisANBODIPYs: first, bis(dipyrromethane) 6b was directly converted to BCOD-bisANBODIPY 8b with four ester groups in 70% yield by treatment with DDQ, *N*,*N*-diisopropylethylamine and BF_3_·OEt_2_. Second, four ester groups of 6b were transformed to four methyl groups by rigorous reduction with LiAlH_4_ in refluxing THF. The rather unstable tetramethyl derivative was then converted to BCOD-bisANBODIPY 7b in 35% yield by treatment with DDQ, *N*,*N*-diisopropylethylamine and BF_3_·OEt_2_. Third, the four ester groups of 6b were removed by treatment with NaOH in ethylene glycol at 170 °C. The unstable α-free derivative obtained was cyanated with chlorosulfonyl isocyanate (CSI)^22^ and then transformed to BCOD-bisANBODIPY 9b with four cyano groups in 5% overall yield from 6b. Bis(dipyrromethane) 6c with two hexyloxyphenyl groups obtained from the reaction of 4c with 5 (75% yield) was converted to BCOD-bisANBODIPY 8c with four ester groups at 80% yield. The thermal treatment of 7b, 8b, 8c and 9b at 200 °C for 2 h or at 250 °C for 30 min *in vacuo* produced almost quantitative yields of B-bisANBODIPYs 10b, 11b, 11c and 12b, respectively (Scheme 2). Both thermal conditions gave similar results.

**Fig. 2:**
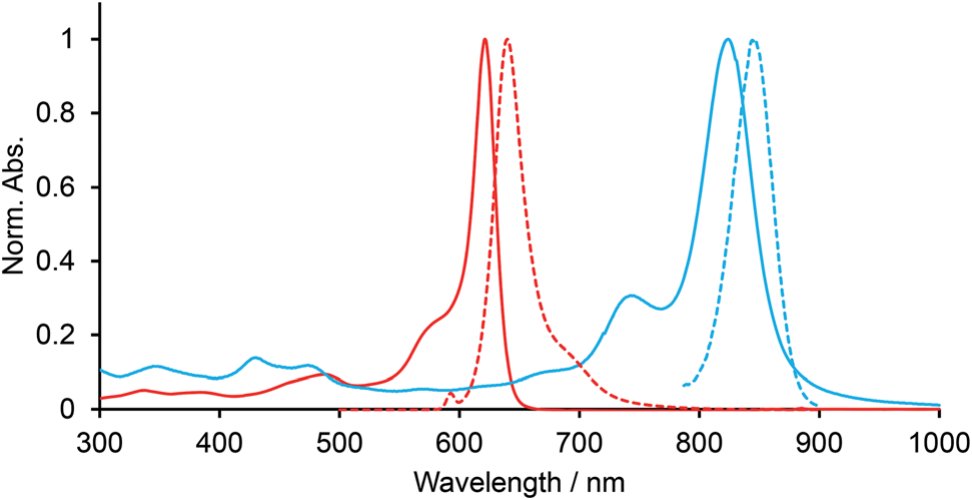
UV-vis-NIR (solid line) and fluorescence (dotted line) spectra of 7b (red line) and 10b (blue line) in CH_2_Cl_2_.

Electronic spectra of BODIPYs in CH_2_Cl_2_ were recorded. Fig. 2 shows the UV-vis-NIR and fluorescence spectra of BCOD-bisANBODIPY 7b and B-bisANBODIPY 10b as representative cases. The spectral shapes of other bisANBODIPYs are almost the same as those of 7b and 10b (see ESI†). The results are listed in Table 1. In the spectra of BCOD-bisANBODIPYs 7a, 7b, 8b and 8c, strong absorption bands with the lowest energy can be observed at *ca.* 625 nm, irrespective of substituent difference between tetramethyl and tetra(ethoxycarbonyl). On the other hand, the absorption band was shifted by *ca.* 20 nm to 646 nm by substitution of four cyano groups. In the case of B-bisANBODIPYs, the absorption bands with the lowest energy shifted by *ca.* 200 nm relative to those of the corresponding BCOD-bisANBODIPYs. The absorption maxima of 10a, 10b, 11b, 11c and 12b were observed at 828, 824, 835, 836 and 900 nm, respectively. In all cases, these large peaks were accompanied by smaller peaks with shorter wavelengths. In the acenaphtho series, differences in absorption maxima with the lowest energy between tetramethyl 10b, tetra(ethoxycarbonyl) 11b and tetracyano 12b were *ca.* 11 and 64 nm. Therefore, the spectrum of tetra(ethoxycarbonyl) 11b resembled that of tetramethyl 10b rather resembling that of tetracyano 14. In the benzo series 1a, 1b and 1c, the opposite tendency was reported.^14^ The spectrum of tetra(ethoxycarbonyl) 1b resembled that of tetracyano 1c. The differences of the absorption maxima between 1a, 1b and 1c were 62 and 10 nm.

**Table 1:** Electronic spectra of bisANBODIPYs in CH_2_Cl_2_

	UV-vis-NIR					Fluorescence	
	nm (*ε* × 10^−4^ M^−1^ cm^−1^) or [relative intensity]					nm (ex. nm)	*Φ* a
7a		486 [0.13]		580 [sh, 0.27]	624 [1.00]	631 (600)	0.40
7b		488 (2.48)		580 (sh, 4.81)	621 (20.1)	640 (580)	0.53
8b	351 (1.83)	422 (1.72)		535 (sh, 3.14)	625 (14.1)	658 (610)	0.07
8c	358 (1.35)	416 (1.82)		540 (3.10)	624 (12.1)	665 (590)	0.10
9b	420 (1.60)	460 (1.78)		—b	646 (8.84)	707 (590)	0.02
10a	348 [0.21]	433 [0.20]	475 [0.19]	746 [0.38]	828 [1.00]	838 (790)	0.08
10b	348 (2.36)	430 (2.50)	472 (2.13)	744 (5.54)	818 (16.9)	846 (800)	0.10
11b	356 (2.34)	443 (1.91)	570 (0.94)	756 (5.18)	835 (15.9)	842 (800)	0.05
11c	366 (2.45)	446 (2.10)	572 (1.00)	758 (5.06)	836 (15.9)	850 (780)	0.04
12b	397 [0.19]	490 [0.14]	624 [0.09]	803 [0.34]	900 [1.00]	—	—

a Absolute quantum yield.

b The shoulder peak was not observed probably due to broadening of the main peak.

**Fig. 3:**
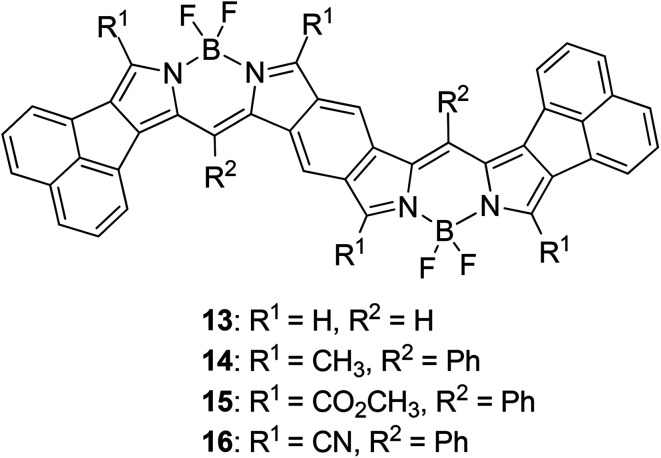
Simplified B-bisANBODIPY structures 13–16 for TD-DFT calculation.

**Fig. 4:**
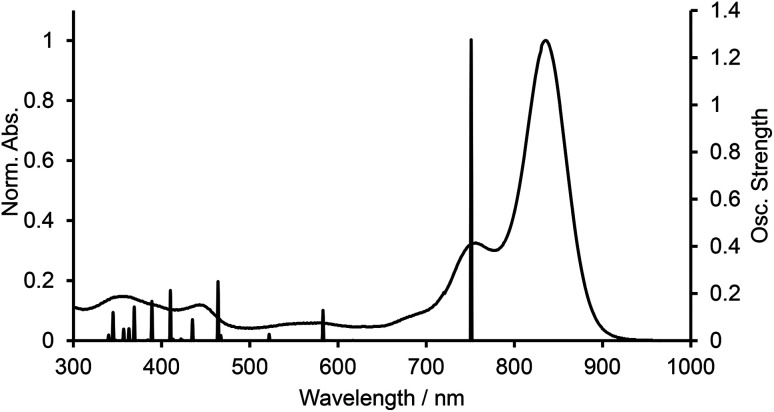
UV-vis-NIR spectra of 11b and calculated spectrum of 15.

We carried out time-dependent density functional theory (TD-DFT) calculation of simplified B-bisANBODIPYs 13, 14, 15 and 16 (Fig. 3) at the level of B3LYP/6-31G+(d) in order to clarify the difference. The representative results are summarized in Table 2, and the typical calculated spectrum of 15 with the corresponding spectrum of 11b is illustrated in Fig. 4. In all cases, the two lower excited states with effective oscillator strength consisted of almost one transition between HOMO and LUMO or between HOMO−2 and LUMO. The energy differences between the two states were 0.5144 eV for 13, 0.6824 eV for 14, 0.3757 eV for 15 and 0.3938 eV for 16. These values are quite larger than those observed for 10a–12b in the UV-vis-NIR spectra. Thus, we concluded that the absorptions accompanying the longest-wavelength maxima were due to vibration. The differences in calculated absorption maxima between tetramethyl 14, tetra(methoxycarbonyl) 15 and tetracyano 16 were 26 and 28 nm. This tendency for calculated absorption maxima can be explained by the electron-withdrawing ability of ester and cyano groups. However, it is not in accordance with the observed tendency: in the case of B-bisANBODIPY series, the difference in absorption energy between tetramethyl and tetra(ethoxycarbonyl) derivatives was small, while that between tetra(ethoxycarbonyl) and tetracyano derivatives was large.

**Table 2:** Two state transitions with the lowest energy and meaningful oscillator strength in TD-DFT calculation for B-bisANBODIPYs

	State^a^	The most contributed transition	Energy		*f*
			eV	nm	
13	1	HOMO → LUMO	1.7310	716	1.5266
	4	HOMO−2 → LUMO	2.2568	549	0.1750
14	1	HOMO → LUMO	1.6445	753	1.449
	4	HOMO−2 → LUMO	2.2887	541	0.1200
15	1	HOMO → LUMO	1.6508	751	1.2759
	4	HOMO−2 → LUMO	2.1253	583	0.1286
16	1	HOMO → LUMO	1.5679	790	1.1908
	4	HOMO−2 → LUMO	2.0192	614	0.1473

a Oscillator strengths of states 2 and 3 are almost zero.

**Fig. 5:**
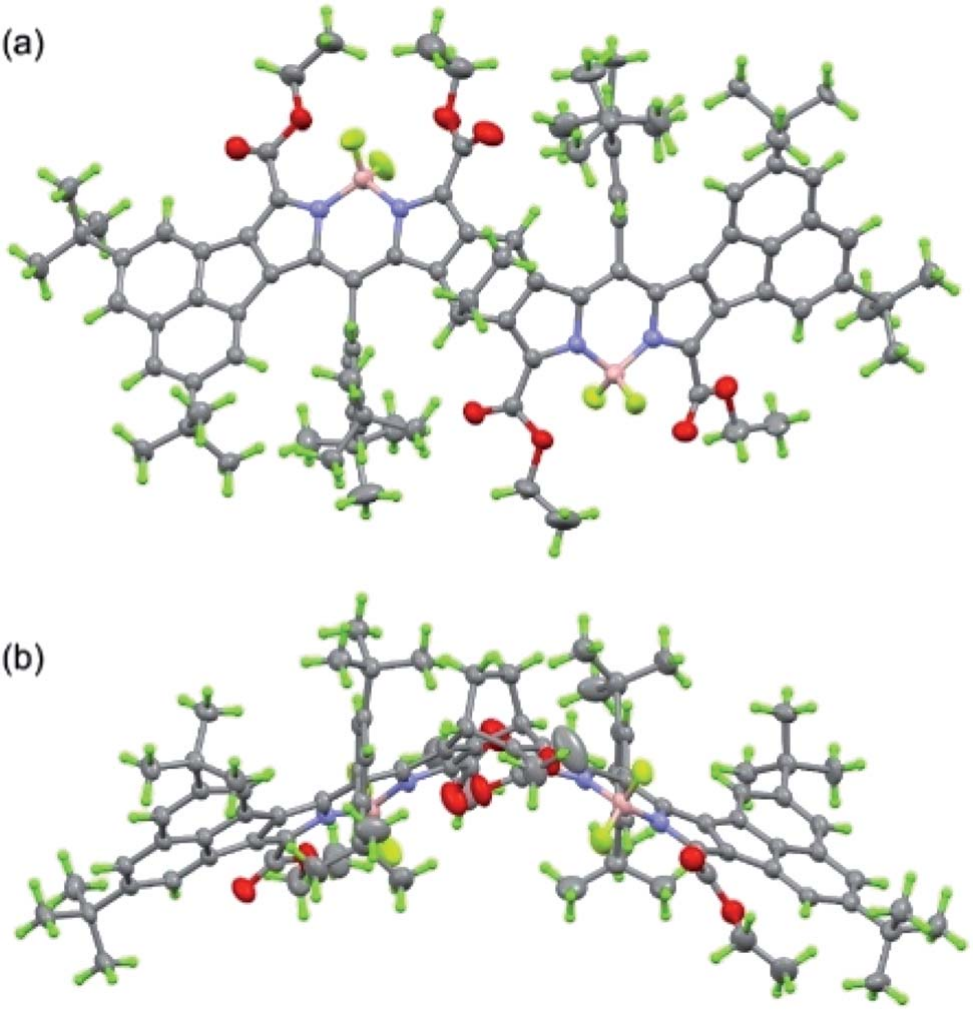
Ortep drawing of 8b (top (a) and side (b) views). Solvent molecules (CHCl_3_ and i-propanol) and disordered substituents with lower occupancies are omitted for clarity.

**Fig. 6:**
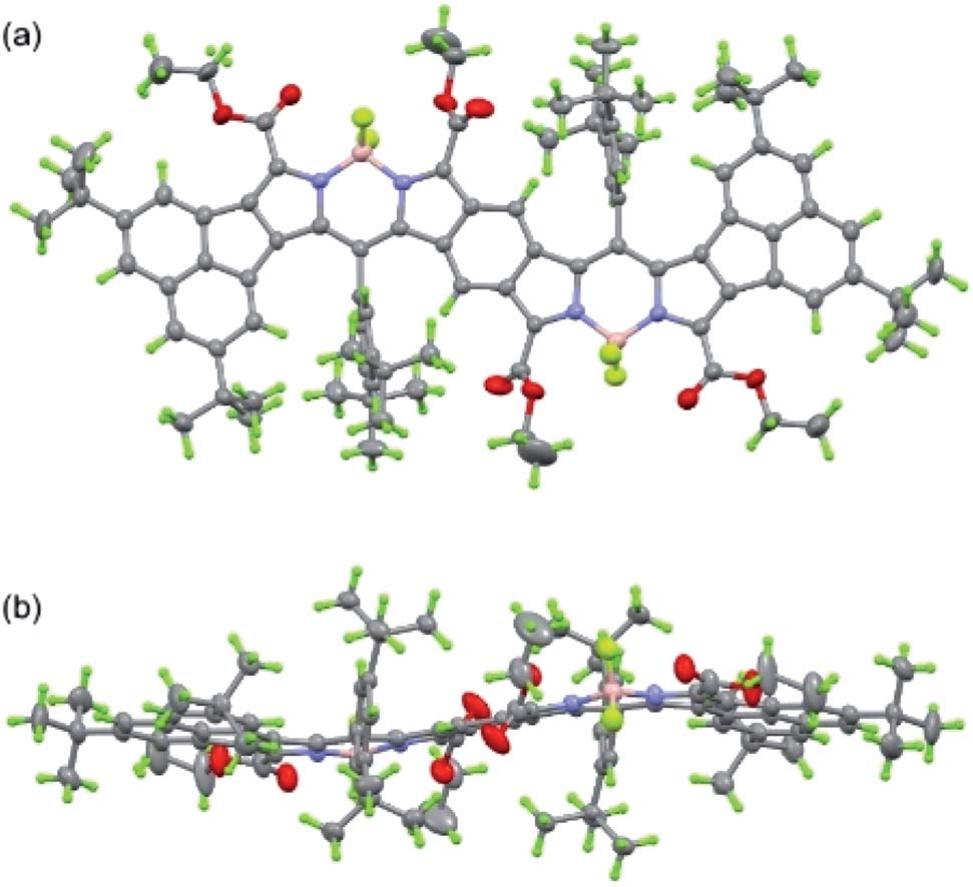
Ortep drawing of 11b (top (a) and side (b) views). The structure was refined by the Platon Squeeze technique. Disordered atoms with lower occupancies and refined acetonitrile are omitted for clarity.

Next, we studied the structure of bisANBODIPYs. Fortunately, single crystals of BCOD- and B-bisANBODIPYs 7b, 8b, 10b, 11b, 11c and 12b were obtained by the solvent–vapor diffusion method: bisANBODIPYs were dissolved in chloroform or dichloromethane and placed under an atmosphere of a poor solvent such as methanol or acetonitrile. In the case of 11b and 12b were dry solvent sets used in order to avoid hydrolysis of a BF_2_ unit (*vide infra*). The crystals obtained were subjected to X-ray analysis. Ortep drawings of 8b and 11b are respectively illustrated in Fig. 5 and 6 as representative, and others are provided in the ESI.† All crystals except for those from 11c were proven to involve co-crystallized solvent molecules in a disordered fashion. B-bisANBODIPY 11c, however, underwent partial hydrolysis during recrystallization from an undried solvent system. The crystal was proven to consist of mono-BF_2_-removed 17 and 11c (17/11c ratio of *ca.* 3 : 1; Fig. 7 and S7 in ESI†). There might be fully-hydrolyzed bisdipyrrin 18 in the crystal. We could not determine the possibility by the X-ray analysis. When the solvent molecules were not properly modeled, the rest of the molecules were refined by the Platon Squeeze technique.

**Fig. 7:**
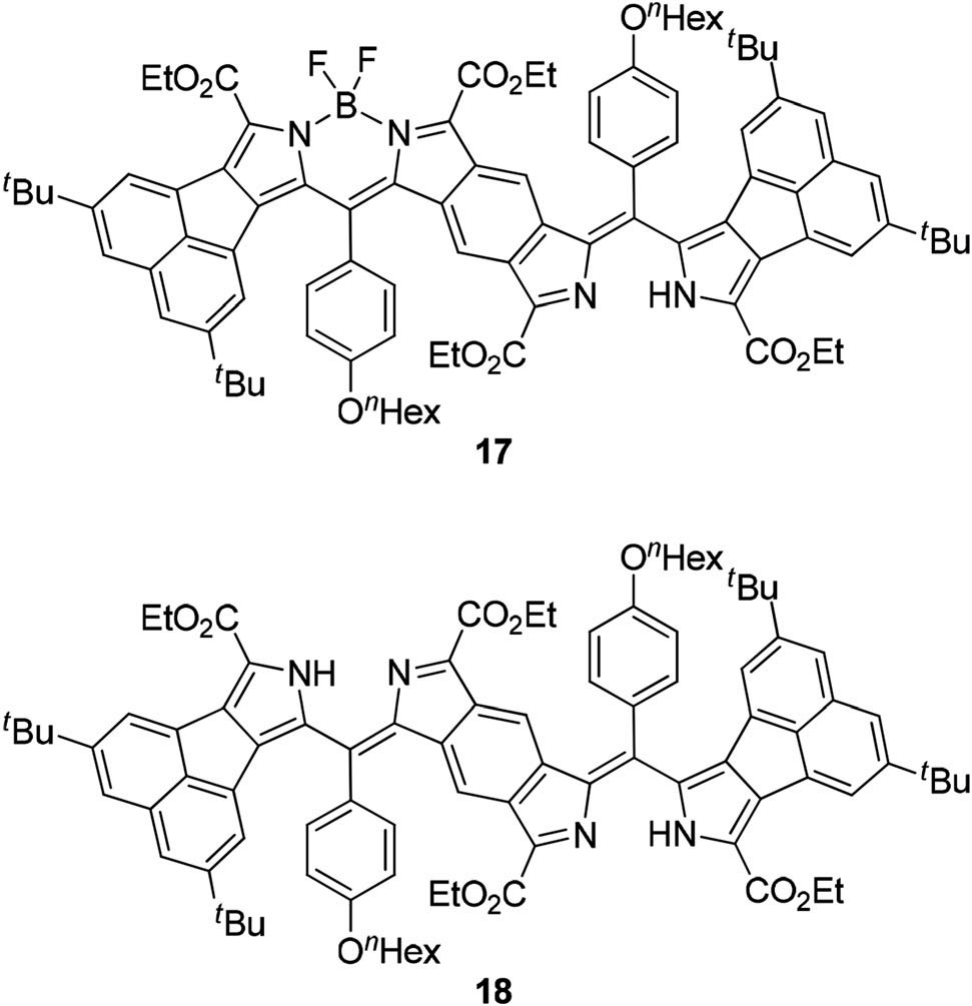
Mono-BF_2_ derivative 17 and bisdipyrrin 18.

In the crystal structure of BCOD-bisANBODIPY 8b, one of the ester parts was disordered. Another ester carbonyl group was directed toward the BF_2_ moiety of BODIPY, even though this conformation was thought to be disadvantageous because of the dipole–dipole interaction. Both ester groups were disordered in one BODIPY part of B-bisANBODIPYs 11c and 17. On the other hand, no disordered ester group was observed in the other BODIPY part, which consisted of 75% dipyrrin and 25% BODIPY. BCOD-bisANBODIPY 8b adopted the gable shape because of the BCOD skeleton and the dihedral angle between the mean planes of twelve BODIPY atoms (121.1(1)°; Table 3). Contrary to almost flat structures of 10b and 12b (see Fig. S6 and S8†), the mixtures of 11c and 17 adopted slightly waved conformation. The dihedral angle between the BODIPY mean planes was 9.64(4)°, and those between the mean planes and benzene moiety were 6.47(8)° and 3.44(7)°. Dihedral angles between the ester and BODIPY planes are also listed in Table 3. The values are rather large probably because of the steric effect of the acenaphtho moiety. This may be the reason for the differences in absorption mentioned earlier. Conjugation of the ester groups to the B-bisANBODIPY chromophore was thought to be poor. Thus, the HOMO and LUMO energy levels were not close enough.

**Table 3:** Dihedral angles in bisANBODIPY tetraesters

	BCOD-bisANBODIPY 8b		B-bisANBODIPY		
			11b^a^	11c and 17^b^	
	BODIPY-1	BODIPY-2	BODIPY	BODIPY-1	BODIPY-2^c^
Benzene	121.1(1)^d^		7.29(8)°	6.47(8)°	3.44(7)°
Proximal ester [occupancy]	29.72(6)° [1.00]	52.09(7)° [1.00]	55.06(3)°	14.91(2)° [0.75]	10.70(2)° [1.00]
				34.52(4)° [0.25]	
Distal ester [occupancy]	26.69(9)° [0.59]	29.63(6)° [1.00]	17.81(4)°	24.76(2)° [0.75]	11.98(3)° [1.00]
	21.32(10)° [0.41]			5.57(5)° [0.25]	

a Compound 11b occupies a special position (−1).

b The crystal consisted of *ca.* 75% of 17 and 25% of 11c.

c This BODIPY part consisted of *ca.* 75% of dipyrrin and 25% of BODIPY.

d The dihedral angle between two BODIPY parts.

**Fig. 8:**
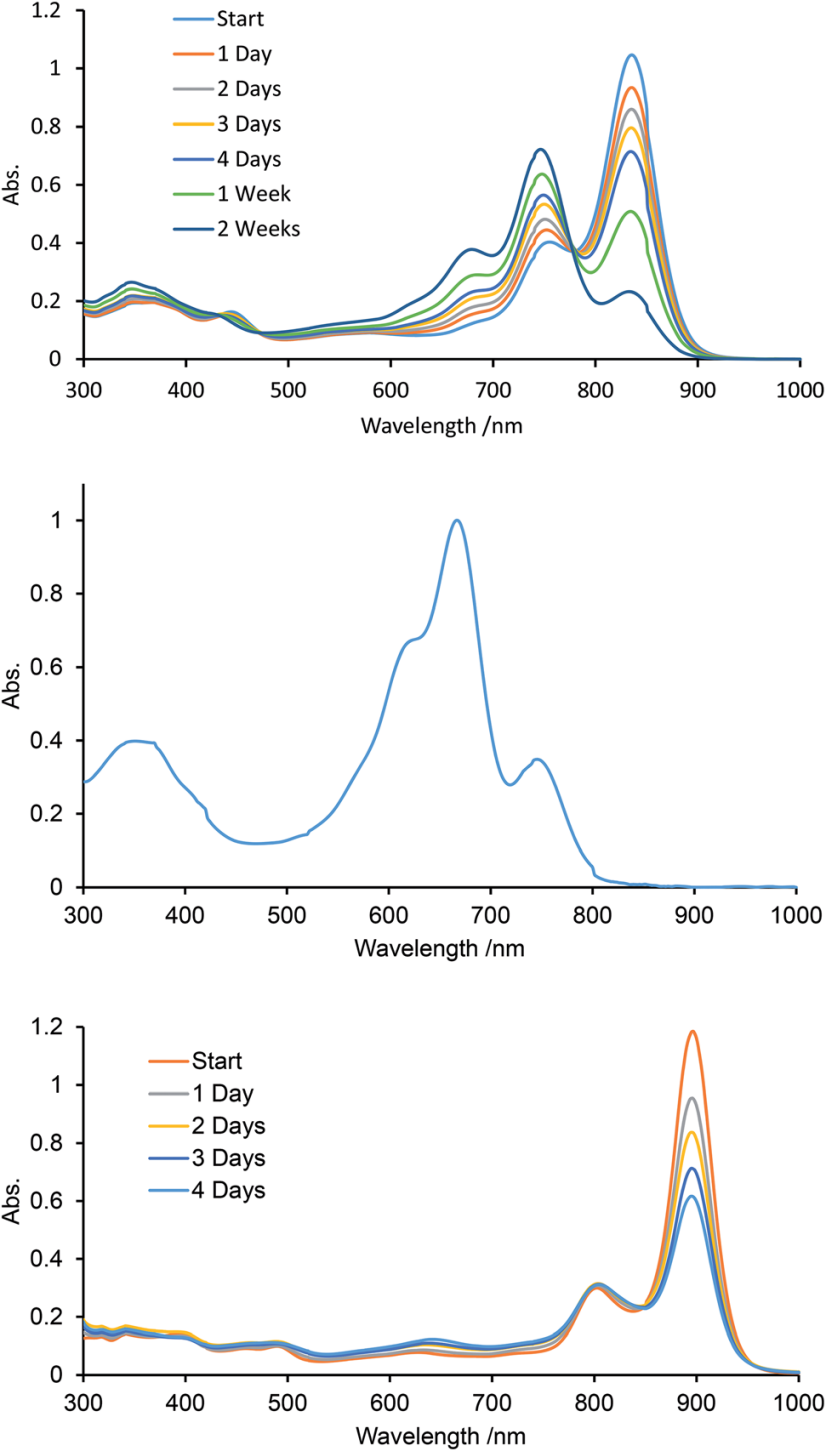
UV-vis-NIR monitoring of 11c (upper: after 1–14 days and middle: after 84 days) and 12b (bottom: after 1–4 days) in spectroscopic-grade CH_2_Cl_2_.

We monitored the UV-vis-NIR spectra of B-bisANBODIPYs 10a, 10b, 11b, 11c and 12b in order to test their stability (Fig. 8, S9 and S10†). We reported a smooth decomposition pattern showing new strong absorption (614 nm) and emission (619 nm) peaks in the case of tetramethyl B-bis(benzoBODIPY) 1a (see Fig. S11 and S12†).^14^ The spectral feature was thought to be due to two benzoBODIPY chromophores, because strength of the absorption was similar to that of 1a. Moreover, the decomposition was suppressed by protection either from oxygen or light. Taking these facts into an account, singlet oxygen was thought to attack the center benzene moiety forming an *endo*-peroxide species, although decomposition of a BODIPY chromophore by singlet oxygen was reported to proceed by initial attack at its C^8^–C^8a^ bond forming a dioxetane species.^23^ Contrary to the B-bis(benzoBODIPY)s 1,^14^ tetracyano derivative 12b was revealed to decompose faster than the other derivatives. As Fig. 8c shows, the absorption spectra of tetracyano derivative 12b only decreased in intensity and no obvious other peak emerged. On the other hand, a new absorption peak slowly appeared at 650 nm in the case of tetramethyl derivative 10a, similarly to the case of B-bis(benzoBODIPY). In the cases of tetra(ethoxycarbonyl) derivatives 11b and 11c, the peaks at 745 nm accompanying with the longest-wavelength absorption maxima (835 and 836 nm) increased (Fig. 8a) and then decreased. After 84 days, the longest-wavelength absorptions completely disappeared and the new large absorptions appeared at 666 and 620 nm (Fig. 8b). The absorption maxima at 745 and 666 nm were determined to be due to mono-BF_2_ derivative 17 and bisdipyrrin 18, respectively, by diagnosis of the X-ray analysis (*vide ante*) and mass spectroscopies (Fig. S14†). This decomposition pattern of tetra(ethoxycarbonyl) derivatives was hydrolysis and was different from that of tetramethyl derivatives. As the single crystals of 12b were obtained from the anhydrous solvent system (*vide ante*), the first decomposition step of 12b was also thought to be hydrolysis. Substitution of electron-withdrawing groups such as cyano and ethoxycarbonyl groups at 3- and 5-positions of 4-bora-3*a*,4*a*-diaza-*s*-indacene skeleton made the BODIPY chromophore labile toward hydrolysis, although the pristine difluoro BODIPY was robust toward hydrolysis under neutral conditions.^24^ Alkyl and/or aryl substituted BODIPYs was only hydrolyzed to dipyrrins under strongly acidic or basic conditions.^25^

Next, B-bisANBODIPYs 10a, 10b, 11b, 11c and 12b were subjected to a cyclic voltammetry experiment (results are summarized in Table 4). In all cases, one reversible oxidation and two reversible reduction peaks were observed. The oxidation half-wave potentials (E_1/2_) of 10b, 11b, 11c and 12b were 0.472, 0.922, 0.944 and 1.273 V, respectively. These values were sufficiently high for resisting oxidation by air. Therefore, the smooth decomposition observed with tetra(ethoxycarbonyl) and tetracyano derivatives could not be ascribed to oxidation. In the case of tetra(ethoxycarbonyl) derivatives 11b and 11c, the decomposition could be due to hydrolysis of the difluorodiazaboridine to dipyrrin moieties by moisture in the spectroscopic-grade dichloromethane. The spectrum after four days was similar to that of the mother liquor obtained in the case of single-crystal synthesis of 11c, and the amounts of 11b and 11c almost did not decrease in the dehydrated solvents. Moreover, mass peaks due to the corresponding mono-hydrolyzed products were observed in the MS spectra of 11b, 11c and 12b. Although the decomposition pathway of 12b was ambiguous at that time, we believed that the first stage of decomposition was hydrolysis.

**Table 4:** CV data for B-bisANBODIPYs^a^

B-bis(ANBODIPY)	E_1/2_/V			
	2nd Ox.	1st Ox.	1st Red.	2nd Red.
10b	0.903	0.472	−1.040	−1.401
11b	—	0.922	−0.528	−0.874
11c	—	0.940	−0.505	−0.854
12b	—	1.273	−0.083	−0.499

a The measurement was performed in CH_2_Cl_2_ by using Pt as electrodes, Ag/Ag^+^ reference electrode, tetrabutylammonium hexafluorophosphate (0.1 M) as a supporting electrolyte, and Fc^+^/Fc as a standard.

## Conclusion

We prepared B-bisANBODIPYs with very strong NIR absorption. The strength of other absorptions in the visible region was less than 15% relative to that of the corresponding absorption maximum at the longest wavelength. Although B-bisANBODIPYs with electron-withdrawing groups were rather labile toward hydrolysis, they proved to be robust toward oxidation by air. Therefore, B-bisANBODIPYs are promising candidates for stable NIR-selective dyes in non-aqueous media such as resins.

## Experimental

### General

Melting points were measured on a Büchi M-565 apparatus and were uncorrected. NMR spectra were obtained with an AL-400 spectrometer at ambient temperature by using CDCl_3_ as a solvent and tetramethylsilane as an internal standard for ^1^H and ^13^C, unless otherwise indicated. IR spectra were obtained on a Thermo Scientific Nicolet iS5 FT-IR spectrometer with an iD5 ATR diamond plate. UV-vis-NIR and fluorescence spectra were recorded on Jasco V-570 and Hitachi F-4500 spectrophotometers, respectively. Absolute quantum yields were measured with a Hamamatsu Photonics C 9920-03G spectrophotometer. Mass spectra were obtained either with a JEOL JMS-700 (EI, 70 eV; FAB^+^, *p*-nitrobenzyl alcohol) or with a JEOL JMS-S3000 (MALDI-TOF). Elemental analyses were performed with a Yanaco MT-5 elemental analyzer at ADRES, Ehime University. Preparative GPC using LC-911 or LC-916 with Jaigel-1H and 2H was performed by Japan Analytical Industry Ltd. Co. Dehydrated solvents were purchased from Kanto Chemical Co. and used without further purification. Commercially available materials were used without further purification.

### X-ray measurement

X-ray measurements of the single crystals at −173 °C were done with Rigaku VariMax Saturn-724 (1.2 kW Mo rotating anode) or a Rigaku VariMax R-AXIS Rapid (1.2 kW Cu rotating anode). Single crystals for X-ray analysis were obtained by the diffusion method: compounds in a soluble solvent were placed in a vapor of a poor solvent. The X-ray diffraction data were processed by using Crystal Clear 1.6.3 or Rapid Auto followed by Crystal Structure Ver. 4.2.5.^26^ Structures were solved by using the processed data with SIR-2004 or SIR-2011,^27^ and then refined by Shelxl-64 (Shelx-2013).^28^ Final structures were validated by Platon CIF check.^29^

### Ethyl 2,5-bis(1,1-dimethylethyl)-9-formyl-8*H*-acenaphtho[1,2-*c*]pyrrole-7-carboxylate (3a)

POCl_3_ (1.9 mL, 23.6 mmol) was added to dry DMF (2.2 mL, 24.5 mmol) at 0 °C under an argon atmosphere, and the mixture was stirred at rt for 30 min. After acenaphthopyrrole 2 (ref. 18) (6.22 g, 16.6 mmol) in dry CH_2_Cl_2_ (115 mL) was added at rt, the mixture was stirred for 90 min. The reaction was quenched by addition of an aqueous saturated solution of sodium acetate (16 mL) and then the mixture was stirred for 30 min. The organic layer was separated and the aqueous layer was extracted with CHCl_3_. The combined organic layer was washed with an aqueous saturated solution of NaHCO_3_, water and brine, dried over anhydrous Na_2_SO_4_, and concentrated *in vacuo*. The residual solid was triturated in hexane to give 6.52 g (16.2 mmol, 98%) of the title compound as a pale yellow powder: mp 230–233 °C (decomp.); ^1^H NMR *δ* 10.20 (s, 1H), 9.55 (br s, 1H), 8.25 (d, *J* 1.4 Hz, 1H), 8.02 (d, *J* 1.4 Hz), 7.82 (d, *J* 1.4 Hz, 1H), 7.78 (d, *J* 1.4 Hz, 1H), 4.55 (d, *J* 7.2 Hz, 2H), 1.60 (t, *J* 7.2 Hz, 3H), 1.50 (s, 18H); ^13^C NMR *δ* 179.1, 161.0, 151.5, 151.2, 137.1, 134.2, 133.7, 130.4, 130.0, 129.7, 126.4, 122.6, 122.2, 122.0, 120.9, 120.1, 61.7, 35.6, 35.5, 31.7, 31.6, 14.7; IR *ν*_max_ 3263, 2963, 1682, 1664 cm^−1^; MS (FAB^+^) *m*/*z* 404 [M^+^ + 1], 403 [M^+^]. Anal. calcd for C_26_H_29_NO_3_ + 1/5H_2_O: C, 76.70; H, 7.28; N, 3.44. Found; C, 76.68; H, 7.24; N, 3.31%.

### Ethyl 2,5-bis(1,1-dimethylethyl)-9-{3′,5′-bis(1′′,1′′-dimethylethyl)benzoyl}-8*H*-acenaphtho[1,2-*c*]pyrrole-7-carboxylate (3b)

To a stirred solution of 3,5-bis(1,1-dimethylethyl)benzoic acid (11.1 g, 45.0 mmol) in dry CH_2_Cl_2_ (30 mL) was added trifluoroacetic anhydride (6.64 mL, 47.4 mmol) at rt under an argon atmosphere, and the mixture was stirred for 15 min. After trifluoroacetic acid (8.53 mL, 111.5 mmol) was added and stirred for an additional 5 min, acenaphthopyrrole 2 (5.94 g, 15.8 mmol) in dry CH_2_Cl_2_ was added and the mixture was stirred for 3 days. After the reaction was quenched with a saturated aqueous solution of NaHCO_3_, the mixture was extracted with CHCl_3_. The organic extract was washed with a saturated aqueous solution of NaHCO_3_, water and brine, dried over Na_2_SO_4_, and concentrated *in vacuo*. The residual solid was triturated in MeOH to give 7.49 g (12.7 mmol; 80%) of the title compound as a pale yellow powder: mp 252–255 °C; ^1^H NMR *δ* 9.56 (br s, 1H), 8.27 (s, 1H), 7.74–7.72 (m, 4H), 7.65 (s, 1H), 6.72 (s, 1H), 4.56 (q, *J* 7.2 Hz, 2H), 1.60 (t, *J* 7.2 Hz, 3H), 1.49 (s, 9H), 1.31 (s, 18H), 1.21 (s, 9H); ^13^C NMR *δ* 188.5, 160.9, 151.6, 151.0, 150.6, 138.7, 135.1, 134.4, 133.9, 130.5, 130.5, 129.5, 126.6, 126.3, 123.1, 122.1, 121.8, 121.7, 121.6, 118.8, 61.4, 35.5, 35.2, 35.0, 31.6, 31.5, 31.3, 14.7; IR *ν*_max_ 3238, 2961, 1720, 1626 cm^−1^; MS (FAB^+^) *m*/*z* 592 [M^+^ + 1], 591 [M^+^]. Anal. calcd for C_40_H_49_NO_3_ + 1/3H_2_O: C, 80.36; H, 8.37; N, 2.34. Found; C, 80.42; H, 8.33; N, 2.37%.

### Ethyl 2,5-bis(1,1-dimethylethyl)-9-(4-(hexyloxy)benzoyl)-8*H*-acenaphtho[1,2-*c*]pyrrole-7-carboxylate (3c)

The reaction of 4-(hexyloxy)benzylic acid (3.35 g, 15.0 mmol) and 2 (1.92 g, 5.00 mmol) was performed according to the procedure described above to give 2.80 g (4.82 mmol, 80%) of the title compound as a gray powder: mp 162–164 °C (decomp.); ^1^H NMR *δ* 9.63 (s, 1H), 8.28 (d, *J* 1.3 Hz, 1H), 7.98 (m, 2H), 7.72 (d, *J* 1.3 Hz, 1H), 7.66 (d, *J* 1.3 Hz, 1H), 7.01 (m, 2H), 6.83 (d, *J* 1.3 Hz, 1H), 4.55 (q, *J* 7.2 Hz, 2H), 4.04 (t, *J* 6.4 Hz, 2H), 1.84 (m, 2H), 1.60 (t, *J* 7.2 Hz, 3H), 1.52–1.46 (m, 11H), 1.40–1.32 (m, 4H), 1.24 (s, 9H), 0.93 (t, *J* 7.2 Hz, 3H); ^13^C NMR *δ* 185.8, 163.1, 160.9, 151.2, 150.7, 134.2, 133.9, 133.8, 131.6, 130.8, 130.7, 130.4, 129.5, 126.2, 121.9, 121.9, 121.7, 121.6, 118.7, 114.6, 68.2, 61.4, 35.5, 35.3, 31.6, 31.5, 31.3, 29.1, 25.7, 22.6, 14.7, 14.0; IR *ν*_max_ 3290, 2950, 2925, 2866, 1687, 1634 cm^−1^; MS (MALDI-TOF) *m*/*z* 582.9, 581.9, 580.9, 579.9; Anal. calcd for C_38_H_45_NO_4_ + 1/7H_2_O: C, 78.37; H, 7.84; N, 2.41. Found: C, 78.36; H, 7.77; N, 2.43%.

### Ethyl 9-acetoxymethyl-2,5-bis(1,1-dimethylethyl)-8*H*-acenaphtho[1,2-*c*]pyrrole-7-carboxylate (4a)

To a stirred solution of 3a (7.26 g, 18.0 mmol) in a mixture of dry THF (180 mL) and dry EtOH (60 mL) was added NaBH_4_ (2.44 g, 64.5 mmol) at 0 °C under an argon atmosphere, and the mixture was allowed to warm to rt. After 2 h, water was added and the mixture was extracted with EtOAc. The organic extract was washed with water and brine, dried over anhydrous Na_2_SO_4_, and concentrated *in vacuo*. The residual solid was triturated in hexane to give a quantitative amount of ethyl 9-hydroxymethyl-2,5-(1,1-dimethylethyl)-8*H*-acenaphtho[1,2-*c*]pyrrole-7-carboxylate as a pale yellow powder: mp 228–231 °C (decomp.); ^1^H NMR *δ* 9.32 (br s, 1H), 8.17 (d, *J* 1.4 Hz, 1H), 7.70 (d, *J* 1.4 Hz, 1H), 7.63 (m, 2H), 5.56 (d, *J* 5.7 Hz, 2H), 4.49 (q, *J* 7.2 Hz, 2H), 2.39 (br s, 1H), 1.58 (t, *J* 7.2 Hz, 3H), 1.46 (s, 18H) IR *ν*_max_ 3432, 3196, 2961, 1662 cm^−1^; MS (MALDI-TOF) *m*/*z* 407.8 [M^+^ + 2], 406.7 [M^+^ + 1], 405.7 [M^+^]. Anal. calcd for C_26_H_31_NO_3_: C, 77.01; H, 7.71; N, 3.45. Found; C, 76.84; H, 7.68; N, 3.43%. The hydroxymethyl derivative (7.30 g, 18.0 mmol) and DMAP (0.338 g, 2.77 mmol) were dissolved in CHCl_3_ (252 mL) under an argon atmosphere and then Ac_2_O (17.1 mL, 180 mmol) was added with stirring. After 30 min, water was added. The organic layer was separated and the aqueous phase was extracted with CHCl_3_. The organic phase was washed with an aqueous saturated solution of NaHCO_3_ (three times), water and brine, dried over anhydrous Na_2_SO_4_, and concentrated. The residual solid was triturated in ether to give 7.44 g (16.6 mmol, 92%) of the title compound as a pale yellow powder: mp 195 °C (decomp.); ^1^H NMR *δ* 9.05 (br s, 1H), 8.18 (d, *J* 1.4 Hz, 1H), 7.73 (d, *J* 1.4 Hz, 1H), 7.70 (d, *J* 1.4 Hz, 1H), 7.66 (d, *J* 1.4 Hz, 1H), 5.40 (s, 2H), 4.49 (q, *J* 7.2 Hz, 1H), 2.16 (s, 3H), 1.58 (t, *J* 7.2 Hz, 3H), 1.47 (s, 9H), 1.46 (s, 9H); ^13^C NMR *δ* 171.4, 161.5, 151.2, 150.9, 134.3, 133.5, 131.4, 131.3, 129.7, 129.6, 123.7, 121.4, 121.3, 120.3, 118.1, 115.4, 60.9, 58.2, 35.5, 35.5, 31.7, 31.6, 20.9, 14.8; IR *ν*_max_ 3257, 2951, 1734, 1673 cm^−1^; MS (FAB^+^) *m*/*z* 447 [M^+^], 388 [M − (CH_3_CO_2_^−^)]. Anal. calcd for C_28_H_33_NO_4_: C, 75.14; H, 7.43; N, 3.13. Found: C, 75.20; H, 7.31; N, 3.20%.

### Ethyl 9-{1′-acetoxy-3,5-bis(1′′,1′′-dimethylethyl)benzyl}-2,5-bis(1,1-dimethylethyl)-8*H*-acenaphtho[1,2-*c*]pyrrole-7-carboxylate (4b)

The reaction of 3b (5.91 g, 10.0 mmol) with NaBH_4_ (1.26 g 33.3 mmol) was carried out according to the procedure described above to afford a quantitative amount of ethyl 9-{1′-hydroxy-{3,5-bis(1′′,1′′-dimethylethyl)benzyl}-2,5-bis(1,1-dimethylethyl)-8*H*-acenaphtho[1,2-*c*]pyrrole-7-carboxylate} as a pale yellow powder: mp 224–226. °C; ^1^H NMR *δ* 9.40 (br s, 1H), 8.19 (s, 1H), 7.64 (s, 1H), 7.49 (s, 1H), 7.46 (s, 1H), 7.40 (s, 1H), 6.54 (s, 1H), 6.10 (s, 1H), 4.48 (q, *J* 7.2 Hz, 2H), 1.57 (t, *J* 7.2 Hz, 3H), 1.46 (s, 9H), 1.25 (s, 18H), 1.22 (s, 9H); ^13^C NMR *δ* 151.6, 150.8, 150.4, 140.1, 134.2, 131.7, 131.5, 129.3, 126.4, 123.1, 122.1, 121.13, 122.10, 119.4, 118.8, 113.8, 72.0, 60.7, 35.5, 35.2, 34.9, 31.7, 31.5, 31.4, 14.9, and three sp^2^ carbon signals were not identified due to overlap; IR *ν*_max_/cm^−1^ 3384 (br), 2951, 1660; MS (MALDI-TOF) *m*/*z* 595.7, 594.9, 593.8. Anal. calcd for C_40_H_51_NO_3_: C, 80.90; H, 8.66; N, 2.36. Found; C, 80.76; H, 8.55; N, 2.35%. Acetylation of the alcohol (5.93 g, 10 mmol) was performed according to the procedure described above to give 4.40 g (6.92 mmol, 69%) of the title compound as a pale yellow powder: mp 187 °C (decomp.); ^1^H NMR *δ* 8.92 (br s, 1H), 8.19 (s, 1H), 7.68 (s, 1H), 7.58 (s, 1H), 7.44 (s, 1H), 7.41 (s, 2H), 7.20 (s, 1H), 7.12 (s, 1H), 4.49 (q, *J* 7.2 Hz, 2H), 2.21 (s, 3H), 1.58 (t, *J* 7.2 Hz, 3H), 1.47 (s, 9H), 1.33 (s, 9H), 1.26 (s, 18H); ^13^C NMR *δ* 170.1, 161.5, 151.4, 151.0, 150.7, 136.5, 134.4, 134.2, 131.6, 131.4, 129.6, 128.2, 127.9, 123.0, 121.9, 121.2, 120.0, 119.2, 114.5, 71.73, 60.8, 35.5, 35.4, 34.9, 31.7, 31.7, 31.4, 21.2, 14.8; IR *ν*_max_ 3286, 2959, 1748, 1658 cm^−1^; MS (FAB^+^) *m*/*z* 636 [M^+^ + 1], 576 [M − (CH_3_CO_2_^−^)]. Anal. calcd for C_42_H_53_NO_4_: C, 79.33; H, 8.40; N, 2.20. Found: C, 79.13; H, 8.51; N, 2.21%.

### Ethyl 9-(1′-acetoxy-4-hexyloxybenzyl)-2,5-bis(1,1-dimethylethyl)-8*H*-acenaphtho[1,2-*c*]pyrrole-7-carboxylate (4c)

The reaction of 3c (2.80 g, 4.82 mmol) with NaBH_4_ (1.13 g, 28.3 mmol) was carried out according to the procedure described above to afford a quantitative amount of ethyl 2,5-bis(1,1-dimethylethyl)-9-(1′-hydroxy-4-hexyloxybenzyl)-8*H*-acenaphtho[1,2-*c*]pyrrole-7-carboxylate as a gray powder: mp 193-197 °C (decomp.); ^1^H NMR *δ* 9.29 (s, br, 1H), 8.19 (m, 1H), 7.64 (m, 1H), 7.50 (m, 1H), 7.47 (m, 2H), 6.92 (m, 2H), 6.44 (m, 1H), 6.06 (d, *J* 4.5 Hz, 1H), 4.50 (q, *J* 7.2 Hz, 2H), 3.93 (t, *J* 6.7 Hz, 2H), 2.43 (d, *J* 4.5 Hz, 1H), 1.77 (m, 2H), 1.59 (t, *J* 7.2 Hz, 3H), 1.48–1.42 (m, 11H), 1.40–1.30 (m, 4H), 1.24 (s, 9H), 0.91 (t, *J* 7.1 Hz, 3H); ^13^C NMR *δ* 159.7, 150.9, 150.6, 134.0, 133.2, 131.7, 131.4, 129.4, 126.3, 121.2, 119.3, 119.0, 114.9, 113.8, 70.9, 67.9, 60.7, 35.5, 35.2, 31.7, 31.5, 31.3, 29.2, 25.7, 22.6, 14.9, 14.0, and five sp^2^ carbon signals were not identified due to overlap; IR *ν*_max_ 3378, 3205, 2956, 2872, 1665 cm^−1^; MS (MALDI-TOF) *m*/*z* 581.9 [M^+^], 564.9 [M^+^ − OH]. Anal. calcd for C_38_H_47_NO_4_: C, 78.45; H, 8.14; N, 2.41. Found: C, 78.39; H, 8.06; N, 2.40%. Acetylation of the alcohol (2.80 g, 4.82 mmol) was performed according to the procedure described above to give 1.96 g (3.11 mmol, 65%) of the title compound as a pale yellow powder: mp 187 °C (decomp.); ^1^H NMR *δ* 9.06 (s, br, 1H), 8.18 (m, 1H), 7.66 (m, 1H), 7.55 (m, 1H), 7.48 (d, *J* 8.7 Hz, 2H), 7.14 (m, 1H), 6.90 (d, *J* 8.7 Hz, 2H), 6.69 (m, 1H), 4.50 (q, *J* 7.2 Hz, 2H), 3.92 (t, *J* 6.4 Hz, 2H), 2.20 (s, 3H), 1.76 (m, 2H), 1.59 (t, *J* 7.2 Hz, 3H), 1.50–1.40 (m, 11H), 1.35–1.30 (m, 4H), 1.29 (s, 9H), 0.90 (t, *J* 6.7 Hz, 3H); ^13^C NMR *δ* 170.2, 161.7, 159.6, 150.9, 150.7, 134.2, 134.2, 131.5, 131.2, 129.5, 129.3, 129.3, 129.3, 128.3, 127.9, 121.3, 121.2, 119.8, 119.5, 114.7, 114.5, 71.2, 67.8, 60.9, 35.5, 35.3, 31.6, 31.5, 31.4, 29.2, 25.7, 22.5, 21.2, 14.8, 14.0; IR *ν*_max_ 3272, 2953, 1695, 1673, 1225 cm^−1^; MS (FAB^+^) *m*/*z* 623 [M^+^], 564 [M − (CH_3_CO_2_^−^)]. Anal. calcd for C_40_H_49_NO_4_: C, 77.01; H, 7.92; N, 2.25. HRMS (FAB^+^): calcd for C_40_H_49_NO_5_, 623.3611. Found 623.3587.

### General procedure for BCOD-fused bis(dipyrromethane)tetracarboxylates

To a stirred solution of acenaphthopyrrole-7-carboxylate 4 (2.0 mmol) and BCOD-fused dipyrrole ester 5 (0.3328 g, 1.05 mmol) in acetic acid (20 mL) was added *p*-toluenesulfonic acid monohydrate (119 mg, 0.56 mmol) at rt, and the mixture was stirred for 3 h. After the reaction was quenched with water, the mixture was extracted with EtOAc. The organic extract was washed with an aqueous saturated NaHCO_3_ (three times), water and brine, dried over anhydrous Na_2_SO_4_, and concentrated *in vacuo*. The residual solid was triturated to give a diastereomeric mixture of BCOD-fused bis(dipyrromethane) derivative as a cream-yellow powder.

Bis(dipyrromethane)tetracarboxylate 6a was obtained in 63% yield (0.724 g, 0.66 mmol) from 4a (0.897 g, 2.0 mmol) and 5 (0.3328 g, 1.05 mmol) as a cream powder: MS (FAB^+^) *m*/*z* 1102.6, 1074.6, 1057.5. Further identification was not performed because of the low solubility.

Bis(3,5-di-*tert*-butylphenyl)-substituted bis(dipyrromethane)tetracarboxylate 6b was obtained in 58% yield (0.429 g, 0.291 mmol) from 4b (0.647 g, 1.02 mmol) and 5 (0.164 g, 0.502 mmol) as a white powder: mp > 250 °C (decomp.); ^1^H NMR *δ* 8.54 (br s, 2H), 8.19 (m, 2H), 8.11 (m, 2H), 7.63 (m, 2H), 7.47 (m, 2H), 7.25 (m, 2H), 7.18 (m, 4H), 6.54 (m, 2H), 5.87 (m, 2H), 4.44–3.90 (m, 10H), 1.46–1.19 (m, 88H); ^13^C NMR (typical signals) *δ* 151.8, 150.9, 150.5, 137.1, 134.1, 131.9, 131.6, 129.4, 129.0, 127.2, 126.5, 122.8, 122.0, 121.1, 119.3, 118.5, 113.9, 113.8, 60.5, 89.9, 43.4, 35.5, 35.2, 34.9, 31.7, 35.2, 34.9, 31.7, 31.5, 31.3, 30.8, 14.6, 14.1; IR *ν*_max_ 3452, 3335, 2960, 2904, 2867, 1697, 1659 cm^−1^; MS (MALDI-TOF) *m*/*z* 1480, 1479, 1453, 1452, 1451. Anal. calcd for C_98_H_118_N_4_O_8_: C, 79.53; H, 8.04; N, 3.79. Found: C, 79.50; H, 8.24; N, 3.76%.

Bis(4-hexyloxyphenyl)-substituted bis(dipyrromethane)tetracarboxylate 6c was obtained in 75% yield (1.69 g, 1.16 mmol) from 4c (1.89 g, 3.0 mmol) and 5 (0.504 g, 1.55 mmol) as a white powder: mp > 250 °C (decomp.); ^1^H NMR *δ* 8.43–8.39 (s, 1H), 8.29–8.18 (m, 4H), 7.64 (m, 2H), 7.51–7.48 (m, 2H), 7.35–7.29 (m, 5H), 6.87–6.82 (m, 4H), 6.37–6.28 (m, 2H), 5.65–5.62 (m, 2H), 4.45–4.35 (m, 6H), 4.15–4.09 (m, 4H), 3.91 (m, 4H), 3.93–3.89 (m, 4H), 1.80–1.72 (m, 4H); ^13^C NMR (diastereomer mixture, typical signals) *δ* 161.6, 158.9, 158.9, 150.9, 150.8, 150.8, 150.7, 150.7, 137.5, 134.1, 134.0, 132.0, 131.9, 131.9, 131.4, 131.3, 130.5, 130.2, 130.2, 130.0, 130.0, 129.9, 129.7, 129.5, 129.5, 128.4, 128.4, 128.3, 127.7, 127.6, 127.5, 127.4, 121.3, 121.1, 119.6, 119.4, 118.8, 115.3, 114.1, 113.8, 113.4, 113.3, 68.0, 67.9, 60.7, 60.7, 60.6, 60.1, 59.9, 43.1, 43.0, 42.6, 35.5, 35.2, 35.2, 35.2, 31.7, 31.6, 31.4, 31.4, 31.2, 31.1, 30.7, 29.2, 27.3, 27.1, 25.7, 22.6, 14.8, 14.8, 14.7, 14.4, 14.3, 14.3, 14.0; IR *ν*_max_ 3434, 3305, 2954, 1692, 1663, 1240 cm^−1^; MS (FAB^+^) *m*/*z* 1455 [M^+^]. HRMS (FAB^+^): calcd for C_94_H_110_N_4_O_10_^+^, 1454.8222. Found: 1454.8247.

### General procedure for BF_2_ complexation of bis(dipyrromethane)

To a stirred solution of BCDO-fused bis(dipyrromethane) (0.5 mmol) in dry CH_2_Cl_2_ (30 mL) was added DDQ (0.379 g, 1.20 mmol) at room temperature in the dark. After the mixture was stirred for 1 h, (i-Pr)_2_EtN (3.0 mL, 20 mmol) was added and the mixture was stirred for 10 min at rt. BF_3_·OEt_2_ (3.0 mL, 23 mmol) was added and the mixture was stirred for 2 h at rt. The reaction was quenched with water, and the mixture was filtered through a Celite pad, which was washed with EtOAc. The filtrate was extracted with EtOAc. The combined organic extract was washed with brine, dried over anhydrous Na_2_SO_4_, and concentrated *in vacuo*. The residual solid was purified by chromatography on silica gel (CH_2_Cl_2_) and preparative GPC.

### BCOD-bisANBODIPY 7a

To a stirred solution of 6a (0.173 g, 0.157 mmol) in dry THF (10 mL), LiAlH_4_ (0.179 g, 4.72 mmol) was added at rt in the dark. The mixture was then heated to reflux for 3 h. After cooling to 0 °C, the reaction was quenched by slow addition of an aqueous saturated sodium tartrate. The mixture was filtered through a Celite pad, which was washed with EtOAc. The filtrate was extracted with EtOAc. The combined organic phase was washed with water and brine, dried over anhydrous Na_2_SO_4_, and concentrated *in vacuo*. The residual material was filtered with CH_2_Cl_2_ through a short silica-gel column, and the filtrate was concentrated *in vacuo* to give 0.051 g (0.0585 mmol, 37%) of tetramethyl bis(dipyrromethane), which was used without further purification. The complexation with BF_2_ was performed according to the general procedure to provide 0.028 g (0.0156 mmol, 27%) of 7a as a reddish purple powder: mp 170 °C (decomp.); ^1^H NMR (CD_2_Cl_2_) *δ* 7.99 (s, 2H), 7.76 (s, 2H), 7.69 (s, 2H), 7.62 (s, 2H), 7.55 (s, 2H), 4.59 (s, 2H), 2.78 (s, 6H), 2.59 (s, 6H), 1.84–1.66 (m, 4H), 1.45 (s, 18H), 1.38 (s, 18H); UV-vis-NIR (CH_2_Cl_2_) *λ*_max_ [relative int.] 486 [0.13], 580 [sh, 0.27], 624 [1.00] nm; florescence (CH_2_Cl_2_) *λ*_max_ 631 nm (excitation 600 nm; *Φ* = 0.4); IR *ν*_max_ 2954, 2926, 2865 cm^−1^; MS (FAB^+^) *m*/*z* 963, 935; HRMS calcd for C_62_H_64_B_2_F_4_N_4_: 962.5253. Found: 962.5238.

### BCOD-bisANBODIPY with four methyl and two 3,5-di-*tert*-butylphenyl group 7b

The reduction of 6b (0.100 g, 0.090 mmol) with LiAlH_4_ (0.170 g, 4.72 mmol) was carried out according to the procedure described above to afford 0.079 g of crude bis(dipyrromethane) with four methyl and two 3,5-di-*tert*-butylphenyl groups, which was used without further purification. Complexation with BF_2_ was performed according to the general procedure to give 0.043 g (0.032 mmol, 35%) of 7b as a reddish purple powder: mp 200 °C (decomp.); ^1^H NMR (CD_2_Cl_2_) *δ* 7.86 (m, 2H), 7.75 (m, 2H), 7.67 (m, 2H), 7.63 (m, 2H), 7.55 (s, 2H), 7.38 (s, 2H), 5.75 (s, 2H), 3.13 (s, 2H), 2.96 (s, 6H), 2.18 (s, 6H), 1.63 (m, 4H), 1.53 (s, 9H), 1.48 (s, 9H), 1.29 (s, 9H), 1.21 (s, 9H); ^13^C NMR *δ* 152.9, 151.1, 151.0, 150.9, 149.8, 149.3, 148.3, 145.2, 141.4, 137.1, 136.3, 135.1, 133.0, 131.9, 131.2, 129.2, 128.9, 128.7, 128.2, 124.9, 124.0, 123.2, 122.5, 120.8, 118.3, 35.5, 35.4, 32.0, 31.6, 31.6, 31.5, 29.7, 28.5, 14.4, 12.5; UV-vis-NIR (CH_2_Cl_2_, *ε* × 10^−4^ M^−1^ cm^−1^) *λ*_max_ 488 (2.48), 580 (sh, 4.81), 621 (20.1) nm; florescence (CH_2_Cl_2_) *λ*_max_ 640 nm (excitation 580 nm; *Φ* = 0.53); IR *ν*_max_ 2957, 1491, 1150, 1012 cm^−1^; MS (FAB^+^) *m*/*z* 1340 [M^+^ + 1], 1311 [M^+^ − C_2_H_4_]; HRMS calcd for C_90_H_104_B_2_F_4_N_4_: 1338.8383. Found: 1338.8337. Anal. calcd for C_90_H_104_B_2_F_4_N_4_ + CH_3_CN + 2H_2_O: C, 78.01; H, 7.90; N, 4.94. Found: C, 77.90; H, 8.40; N, 4.99%. Single crystals were obtained by diffusion of hexane into a chloroform solution of 7b. CCDC No. 1821923.

### BCOD-bisANBODIPY with four ethoxycarbonyl and two 3,5-di-*tert*-butylphenyl groups 8b

The complexation of 6b (0.213 g, 0.14 mmol) with BF_2_ was performed according to the general procedure to give 0.155 g (0.0986 mmol, 70%) of 8b as a reddish purple powder: mp 230 °C (decomp.); ^1^H NMR *δ* 8.21 (d, *J* 1.4 Hz, 2H), 7.87 (m, 2H), 7.70 (m, 2H), 7.69 (m, 2H), 7.46 (m, 2H), 7.31 (m, 2H), 5.70, (d, *J* 1.4 Hz, 2H), 4.61, (m, 4H), 4.35 (q, *J* 7.1 Hz, 4H), 3.84 (m, 2H), 1.60 (m, 4H), 1.56 (t, *J* 7.1 Hz, 6H), 1.51 (s, 18H), 1.45 (s, 18H), 1.43 (t, *J* 7.1 Hz, 6H), 1.22 (s, 18H), 1.20 (s, 18H); ^13^C NMR *δ* 161.1, 160.3, 153.3, 151.2, 150.8, 150.7, 150.7, 1506, 147.8, 140.6, 139.1, 139.0, 137.2, 133.9, 133.5, 131.6, 131.2, 130.8, 129.5, 129.2, 126.0, 125.6, 125.2, 124.1, 122.6, 122.4, 121.5, 61.9, 61.5, 35.54, 35.52, 35.2, 35.1, 32.9, 32.0, 31.6, 31.4, 31.2, 28.5, 14.4, 13.9; UV-vis-NIR (CH_2_Cl_2_, *ε* × 10^−4^ M^−1^ cm^−1^) *λ*_max_ 351 (1.83), 422 (1.72), 535 (sh, 3.14), 625 (14.1) nm; fluorescence (CH_2_Cl_2_) *λ*_max_ 658 nm (excitation 610 nm; *Φ* = 0.07); IR *ν*_max_ 2955, 2903, 2867, 1750, 1702 cm^−1^; MS (FAB^+^) *m*/*z* 1594 [M + Na^+^], 1572 [M^+^ + 1], 1552 [M − F^−^], 1543 [M + H^+^ − C_2_H_4_]; Anal. calcd for C_98_H_112_B_2_F_4_N_4_O_8_: C, 74.90; H, 7.18; N, 3.57. Found; C, 74.94; H, 7.39; N, 3.51%. Single crystals were obtained by diffusion of 2-propanol into a chloroform solution of 8b. CCDC No. 1821927.

### BCOD-bisANBODIPY with four ethoxycarbonyl and two 4-hexyloxyphenyl groups 8c

The complexation of 6c (0.400 g, 0.27 mmol) was performed according to the general procedure to afford 0.346 g (0.22 mmol, 80%) of 8c as a reddish purple powder: mp 217 °C (decomp.); ^1^H NMR *δ* 8.25 (d, *J* 1.2 Hz, 2H), 7.71 (s, 4H), 7.48 (m, 2H), 7.40 (m, 2H), 7.29 (m, 2H), 7.22 (m, 2H), 5.68 (m, 2H), 4.64 (q, *J* 7.2 Hz, 4H), 4.56–4.42 (m, 4H), 4.26–4.15 (m, 4H), 3.42 (br s, 2H), 1.97 (m, 4H), 1.61 (m, 4H), 1.59 (t, *J* 7.2, 6H), 1.54 (s, 18H), 1.49 (t, *J* 7.0 Hz, 6H), 1.48–1.42 (m, 12H), 1.23 (s, 18H) 0.98 (t, *J* 7.2 Hz, 6H); ^13^C NMR *δ* 161.3, 161.0, 160.9, 151.9, 151.3, 150.9, 148.9, 140.0, 139.4, 139.2, 138.8, 133.5, 131.6, 131.3, 130.8, 130.2, 129.5, 129.1, 127.3, 126.8, 124.9, 124.5, 122.9, 122.6, 116.7, 116.2, 68.2, 62.0, 61.8, 35.6, 35.5, 33.2, 31.7, 31.6, 31.6, 31.2, 29.4, 27.4, 26.0, 22.7, 14.4, 14.1; UV-vis-NIR (CH_2_Cl_2_, *ε* × 10^−4^ M^−1^ cm^−1^) *λ*_max_ 358 (1.35), 416 (1.82), 540 (3.10), 624 (12.1) nm; fluorescence (CH_2_Cl_2_) *λ*_max_ 665 nm (excitation 590 nm; *Φ* = 0.10); IR *ν*_max_ 2654, 2933, 2867, 1748, 1704 cm^−1^; MS (FAB^+^) *m*/*z* 1586 [M + K^+^], 1570 [M + Na^+^], 1528 [M − F^−^], 1519 [M + H^+^ − C_2_H_4_]. Anal. calcd for C_94_H_104_B_2_F_4_N_4_O_10_ + C_6_H_14_: C, 73.52; H, 7.28; N, 3.43. Found C, 73.82; H, 7.00; N, 3.72%.

### BCOD-bisANBODIPY with four cyano and two 3,5-di-*tert*-butylphenyl groups 9b

To a suspension of 6b (0.4415 g, 0.29 mmol) in ethylene glycol (10 mL) was added NaOH (0.28 g, 7.0 mmol) under an argon atmosphere. The mixture was heated at 170 °C for 3 h in the dark. After the mixture was cooled to rt, water was added. The mixture was extracted with EtOAc. The organic extract was washed with water and brine, dried over anhydrous Na_2_SO_4_, and concentrated *in vacuo*. The residual solid was chromatographed on silica gel (40% EtOAc/hexane) to give the compound without esters, which was directly used without identification. This material was dissolved in a mixture of dry DMF (11 mL) and dry CH_3_CN (4.5 mL) and then cooled to −50 °C. CSI (0.17 mL, 1.97 mmol) in acetonitrile (2 mL) was added dropwise at the same temperature. The mixture was stirred at −50 °C for 1.5 h and then at rt overnight. The reaction was quenched by addition of water, and the mixture was extracted with EtOAc. The organic extract was washed with an aqueous saturated NaHCO_3_ solution, water and brine, dried over anhydrous Na_2_SO_4_, and concentrated *in vacuo*. The residual material was chromatographed on silica gel (40% EtOAc/hexane) to give the tetracyano compound, which was directly used without identification. The complexation of the tetracyanide with BF_2_ was achieved through the general procedure. The target BCOD-fused tetracyano bis(acenaphthoBODIPY) 9 was obtained in 5% yield (21 mg, 0.015 mmol) as a reddish purple powder: mp 200 °C (decomp.) ^1^H NMR *δ* 8.10 (d, *J* 1.1 Hz, 2H), 8.00 (t, *J* 1.8 Hz, 2H), 7.86 (d, *J* 1.2 Hz, 2H), 7.83 (d, *J* 1.2 Hz, 2H), 7.51 (t, *J* 1.8 Hz, 2H), 7.32 (t, *J* 1.8 Hz, 2H), 5.90 (s, 2H), 3.54 (2H), 1.53 (s, 18H), 1.46 (m, 20H), 1.29 (m, 20H), 1.20 (s, 18H); ^13^C NMR *δ* 155.0, 152.2, 152.1, 151.9, 150.6, 149.3, 148.7, 142.2, 147.4, 133.1, 132.8, 132.6, 131.5, 130.0, 129.5, 127.5, 127.1, 126.7, 125.4, 124.7, 124.1, 122.0, 120.7, 119.2, 116.6, 111.5, 110.3, 35.7, 35.6, 35.6, 35.4, 32.3, 31.9, 31.5, 31.5, 31.3, 28.0; IR *ν*_max_ 2957, 2225, 1490, 1129, cm^−1^; UV-vis-NIR (CH_2_Cl_2_) [relative int.] *λ*_max_ 417 [0.17], 461 [0.20], 570 [sh, 0.36], 647 [1.00] nm; fluorescence (CH_2_Cl_2_) *λ*_max_ 707 nm (excitation 590 nm; *Φ* = 0.02); IR *ν*_max_ 2957, 2225, 1490, 1129 cm^−1^; MS (FAB^+^) *m*/*z* 1383, 1364, 1355, 1338, 1336. Anal. calcd for C_90_H_92_B_2_F_4_N_8_ + 3H_2_O: C, 75.20; H, 6.87; N, 7.80. Found; C, 75.28; H, 6.69; N, 7.67%.

### General procedure for thermal conversion of BCOD-bisANBODIPY to B-bisANBODIPY

BCOD-bisANBODIPY was weighed in a microtube, which was placed in a test tube. The test tube was evacuated by an oil rotary pump and placed in a glass tube oven, which was pre-heated at the indicated temperature. After the indicated time, the test tube was removed and cooled to rt. The conversion was quantitative.

### B-bisANBODIPY 10a

The conversion of 7a (5.0 mg) was performed at 200 °C for 2 h to afford 4.9 mg (94%) of 10a as a black powder: UV-vis-NIR [CH_2_Cl_2_, relative int.] *λ*_max_ 348 [0.21], 433 [0.20], 475 [0.19], 746 [0.38], 828 [1.00] nm; FL (CH_2_Cl_2_) *λ*_max_, 838 nm; MS (MALDI-TOF) *m*/*z* 937.4, 936.3, 935.3; HRMS calcd for C_60_H_60_B_2_F_4_N_4_: Me 934.4940. Found: Ma 934.4942.

### B-bisANBODIPY 10b

The conversion of 7b (4.3 mg) was performed at 200 °C for 2 h to afford 4.1 mg (97%) of 10b as a black powder: mp > 300 °C (decomp.); ^1^H NMR *δ* 7.93 (t, *J* = 1.7 Hz, 2H), 7.69 (d, *J* = 1.3 Hz, 2H), 7.58 (d, *J* = 1.3 Hz, 2H), 7.55 (d, *J* = 1.3 Hz, 2H), 7.47 (d, *J* = 1.8 Hz, 4H), 5.90 (d, *J* = 1.3 Hz, 2H), 5.77 (s, 2H), 2.93 (s, 6H), 2.68 (s, 6H), 1.45 (s, 18H), 1.38 (s, 36H), 1.17 (s, 18H); ^13^C NMR *δ* 158.3, 153.5, 150.9, 150.9, 143.0, 141.1, 136.1, 135.4, 134.0, 133.7, 133.3, 132.3, 131.8, 131.4, 129.9, 129.4, 127.0, 123.3, 123.1, 122.4, 122.0, 119.9, 117.4, 117.2, 35.5, 35.4, 35.3, 31.9, 31.7, 31.6, 14.1, 12.6; IR *ν*_max_ 2953, 1506, 1149, 1105 cm^−1^; UV-vis-NIR (CH_2_Cl_2_, *ε* × 10^−4^ M^−1^ cm^−1^) *λ*_max_ 348 (2.36), 430 (2.50), 472 (2.13), 744 (5.54), 818 (16.9) nm; FL (CH_2_Cl_2_) *λ*_max_, 846 nm; MS (FAB^+^) *m*/*z* 1311 (M^+^ + 1); HRMS calcd for C_88_H_100_B_2_F_4_N_4_: 1310, 8070. Found: 1310.8045. Anal. calcd for C_88_H_100_B_2_F_4_N_4_ + H_2_O: C, 79.51; H, 7.73; N, 4.21. Found; C, 79.27; H, 7.93; N, 4.29%. Single crystals were obtained by diffusion of acetonitrile into a dichloromethane solution of 10b. CCDC No. 1821919.

### B-bisANBODIPY 11b

The conversion of 8b (5.0 mg) was performed at 250 °C for 30 min to afford 4.9 mg (98%) of 11b as a black powder: mp > 300 °C (decomp.); ^1^H NMR *δ* 8.34 (m, 2H), 7.91 (m, 2H), 7.68 (m, 4H), 7.43 (d, *J* 1.7 Hz, 4H), 6.09 (s, 2H), 5.86 (d, *J* 1.2 Hz, 2H), 4.67–4.58 (m, 8H), 1.62–1.58 (m, 12H), 1.46 (s, 18H), 1.36 (s, 36H), 1.18 (s, 18H); ^13^C NMR *δ* 161.1, 159.7, 153.5, 151.1, 150.7, 147.6, 143.6, 142.8, 138.7, 134.7, 134.1, 132.9, 132.8, 132.3, 131.4, 130.1, 129.5, 129.2, 124.3, 124.0, 123.3, 122.9, 122.6, 122.5, 121.9, 119.7, 63.0, 61.5, 35.5, 35.5, 35.4, 35.3, 32.0, 31.9, 31.8, 31.6, 31.4, 31.4, 31.3, 14.5, 13.9; UV-vis-NIR (CH_2_Cl_2_, *ε* × 10^−4^ M^−1^ cm^−1^) *λ*_max_ 356 (2.34), 443 (1.91), 570 (0.94), 756 (5.18), 835 (15.9) nm; fluorescence (CH_2_Cl_2_) *λ*_max_ 846 nm; IR *ν*_max_ 2952, 2905, 2868, 1726, 1705 cm^−1^; MS (MALDI-TOF) *m*/*z* 1543.8 [M^+^ + 1], 1496.8 [M − BF_2_ + H + 1]; Anal. calcd for C_96_H_108_B_2_F_4_N_4_O_8_: C, 74.70; H, 7.05; N, 3.63. Found: C, 74.71; H, 7.27; N, 3.56%. Single crystals were obtained by diffusion of acetonitrile into a chloroform solution of 11b. CCDC No. 1821922.

### B-bisANBODIPY 11c

The conversion of 8c (13.0 mg) was performed at 250 °C for 30 min to afford 12.6 mg (97%) of 11c as a black powder: mp > 300 °C (decomp.); ^1^H NMR *δ* 8.33 (d, *J* 1.3 Hz, 2H), 7.69 (m, 2H), 7.66 (m, 2H), 7.51 (m, 4H), 7.33 (m, 4H), 6.62 (s, 2H), 5.72 (s, 2H), 4.69–4.56 (m, 8H), 4.23 (m, 4H), 1.98 (m, 4H), 1.68–1.60 (m, 10H), 1.55 (t, *J* 7.3 Hz, 6H), 1.47 (m, 26H), 1.22 (s, 18H), 1.00 (t, *J* 7.0 Hz, 6H); ^13^C NMR *δ* 161.2, 160.8, 159.9, 151.1, 150.7, 148.1, 143.5, 141.5, 138.7, 134.1, 132.8, 132.5, 132.4, 132.3, 131.1, 130.6, 129.9, 129.2, 126.7, 123.7, 123.3, 122.7, 122.6, 122.1, 120.0, 116.7, 68.2, 63.1, 61.5, 35.5, 35.4, 31.7, 31.7, 31.6, 31.2, 31.1, 29.5, 22.7, 14.7, 14.1; UV-vis-NIR (CH_2_Cl_2_, *ε* × 10^−4^ M^−1^ cm^−1^) *λ*_max_ 366 (2.45), 446 (2.10), 572 (1.00), 758 (5.06), 836 (15.9) nm; fluorescence (CH_2_Cl_2_) *λ*_max_ 844 nm; IR *ν*_max_ 2952, 2867, 1722, 1696 cm^−1^; MS (MALDI-TOF) *m*/*z* 1519.6 [M^+^], 1500.6 [M − F^−^], 1471.6 [M^+^ − BF_2_]. Anal. calcd for C_92_H_100_B_2_F_4_N_4_O_10_: C, 72.72; H, 6.63; N, 3.69. Found; C, 72.57; H, 6.62; N, 3.85%. Single crystals were obtained by diffusion of 2-propanol into a chloroform solution of 11c. CCDC No. 1821920.

### B-bisANBODIPY 12b

The conversion of 9 (6.9 mg) was performed at 250 °C for 5 h to afford 6.8 mg (100%) of 12b as a black powder: mp > 300 °C (decomp.); ^1^H NMR *δ* 8.11 (m, 2H), 8.09 (s, 2H), 7.83 (s, 2H), 7.82 (s, 2H), 7.45, (m, 4H), 6.11 (s, 2H), 5.98 (s, 2H), 1.47 (s, 18H), 1.41 (s, 36H), 1.19 (s, 18H); ^13^C NMR (typical signals) *δ* 171.7, 168.6, 155.0, 152.0, 133.4, 131.7, 35.8, 35.6, 35.5, 35.4, 31.7, 31.5, 31.4; UV-vis-NIR [CH_2_Cl_2_, relative int.] *λ*_max_ 397 [0.19], 490 [0.14], 624 [0.09], 803 [0.34], 900 [1.00] nm; IR *ν*_max_ 2957, 2905, 2869, 2221 cm^−1^; MS (FAB^+^) *m*/*z* 1355 [M^+^ + 1], 1336 [M − F^−^], 1308 [M − BF_2_ + H + H^+^], 1307 [M − BF_2_ + H^+^]. Anal. calcd for C_88_H_88_B_2_F_4_N_8_ + 5/2H_2_O: C, 75.48; H, 6.69; N, 8.00. Found: C, 75.54; H, 6.50; N, 8.00%. Single crystals were obtained by diffusion of dry acetonitrile into a dry chlorobenzene solution of 12b. CCDC No. 1821205.

## Conflicts of interest

There are no conflicts to declare.

## Supplementary Material

RA-008-C8RA01694A-s001

RA-008-C8RA01694A-s002

RA-008-C8RA01694A-s003
