# Supplementary material for: Benzene-fused bis(acenaphthoBODIPY)s, stable near-infrared-selective dyes
Source: RSC Adv. 2018 Apr 16;8(25):14072–83. doi: 10.1039/c8ra01694a (PMC9079909; doi:10.1039/c8ra01694a)
Supplement: RA-008-C8RA01694A-s001 [file RA-008-C8RA01694A-s001.pdf]

Electronic Supplementary Material (ESI) for RSC Advances.
This journal is © The Royal Society of Chemistry 2018

Benzene-fusing bis(acenaphthoBODIPY)s as a stable near-infrared-selective dye

Hidemitsu Uno,*[,1] Takayuki Honda,[1] Manami Kitatsuka,[1] Shogo Hiraoka,[1] Shigeki Mori,[2] Tetsuo Okujima,[1] Masayoshi Takase,[1] and Takahiro Nakae[1,3]

[1] Department of Chemistry and Biology, Graduate School of Science and Engineering, Ehime University, 2-5 Bunkyo-cho, Matsuyama 790-8577, Japan

[2] Division of Material Science, Advanced Research Support Center, Ehime University, 2-5 Bunkyo-cho, Matsuyama 790-8577, Japan

[3] Present address: Institute of Advanced Energy, Kyoto University, Gokasyo, Uji 711-0011, Japan

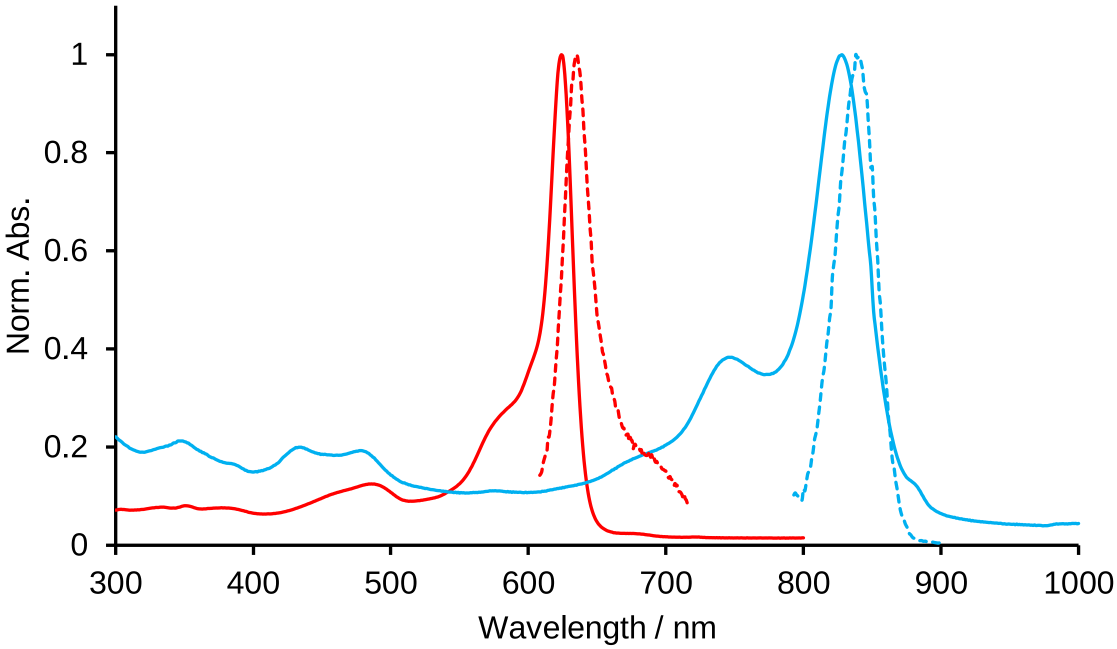

Figure S1. UV-vis-NIR (solid line) and fluorescence (dotted line) spectra of **7b** (red line) and **10b** (blue line) in CH_2Cl_2

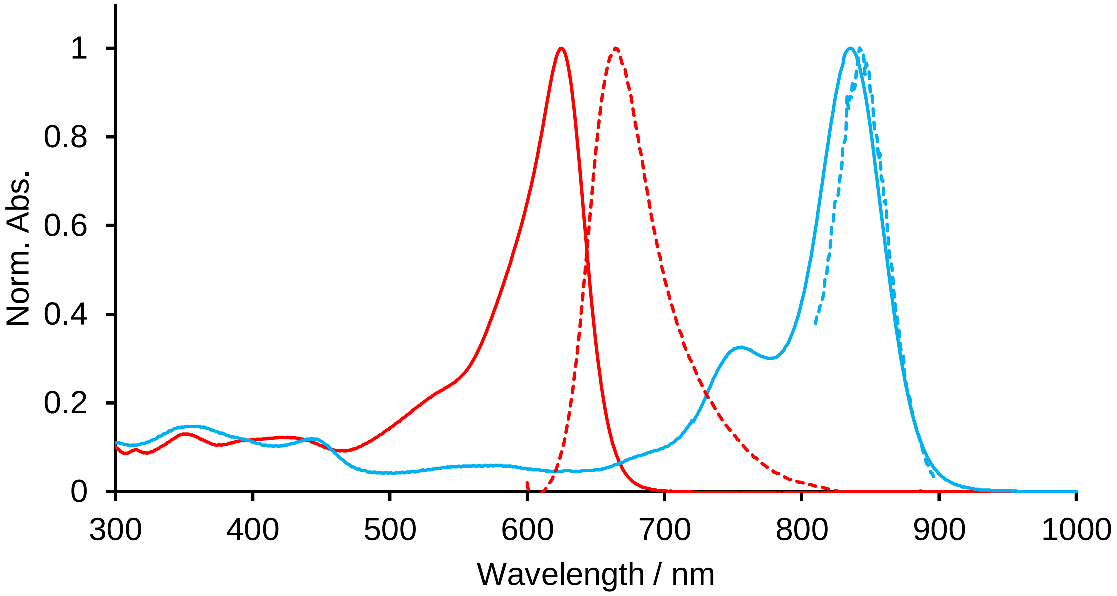

Figure S2. UV-vis-NIR (solid line) and fluorescence (dotted line) spectra of **8b** (red line) and **11b** (blue line) in CH_2Cl_2

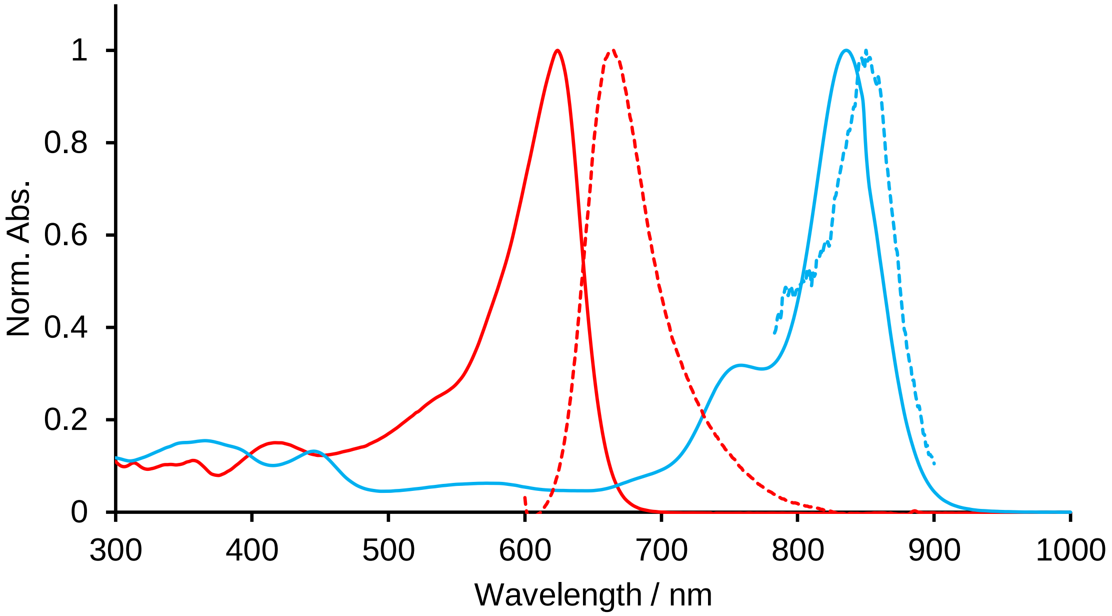

Figure S3. UV-vis-NIR (solid line) and fluorescence (dotted line) spectra of **8c** (red line) and **11c** (blue line) in CH_2Cl_2

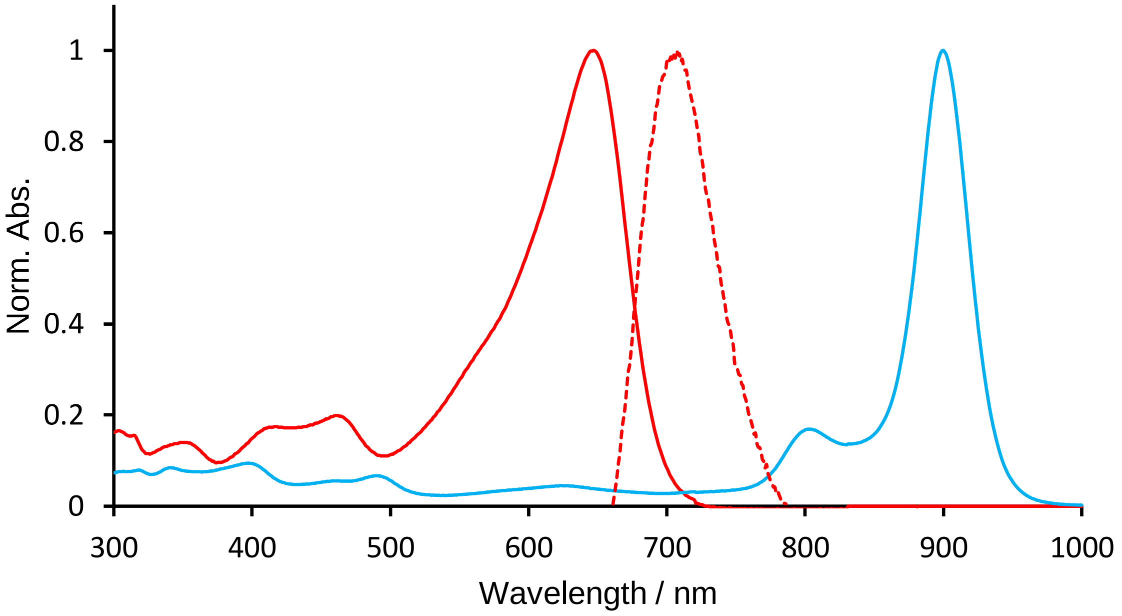

Figure S4. UV-vis-NIR (solid line) and fluorescence (dotted line) spectra of **9b** (red line) and **12b** (blue line) in CH_2Cl_2. Fluorescence of **12b** could not be measured due to the wavelength limitation of machine.

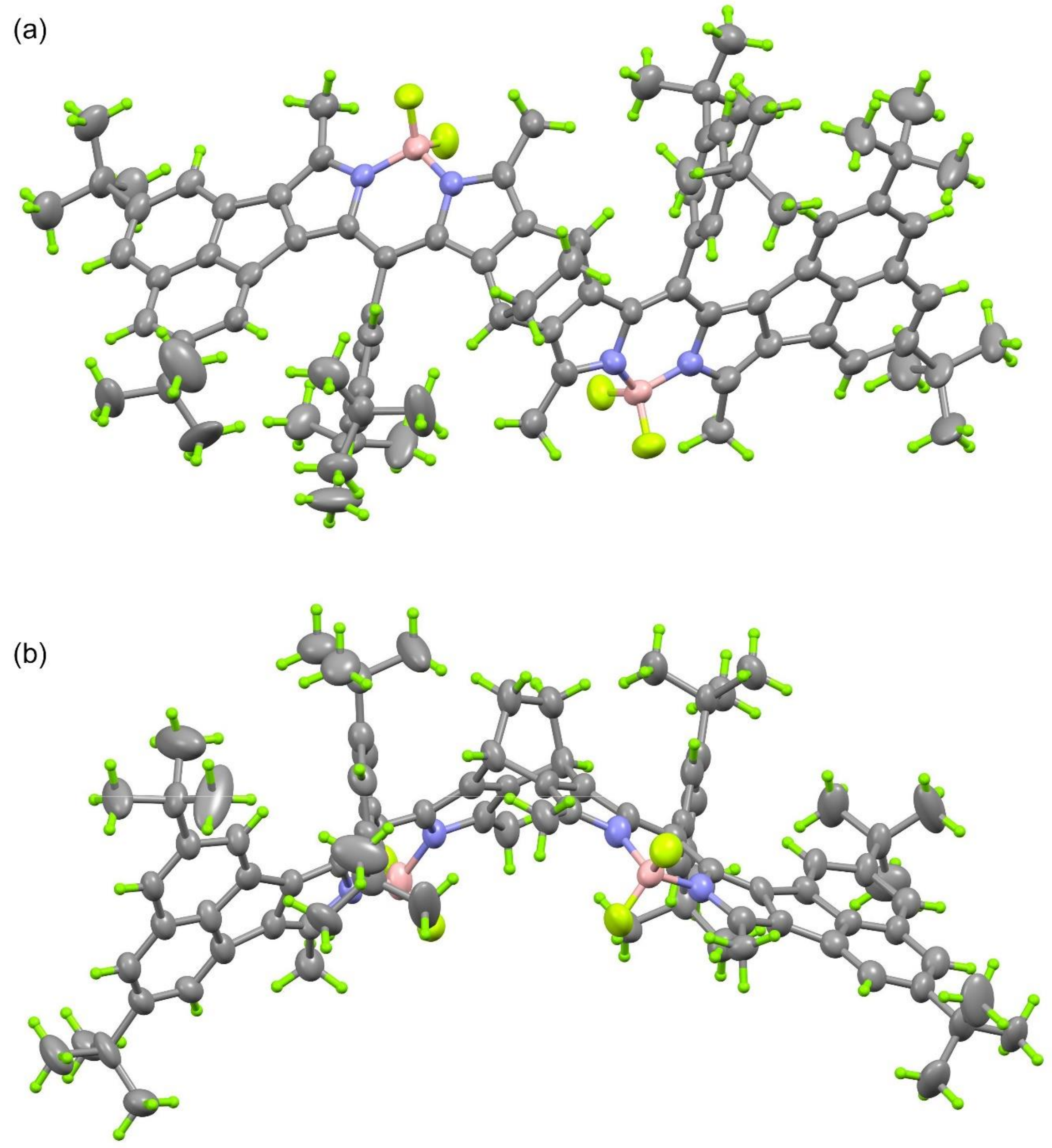

Figure S5. Ortep drawing of **7b** (top (a) and side (b) views). Solvent molecules and disordered substituents with less occupancy are omitted for clarity.

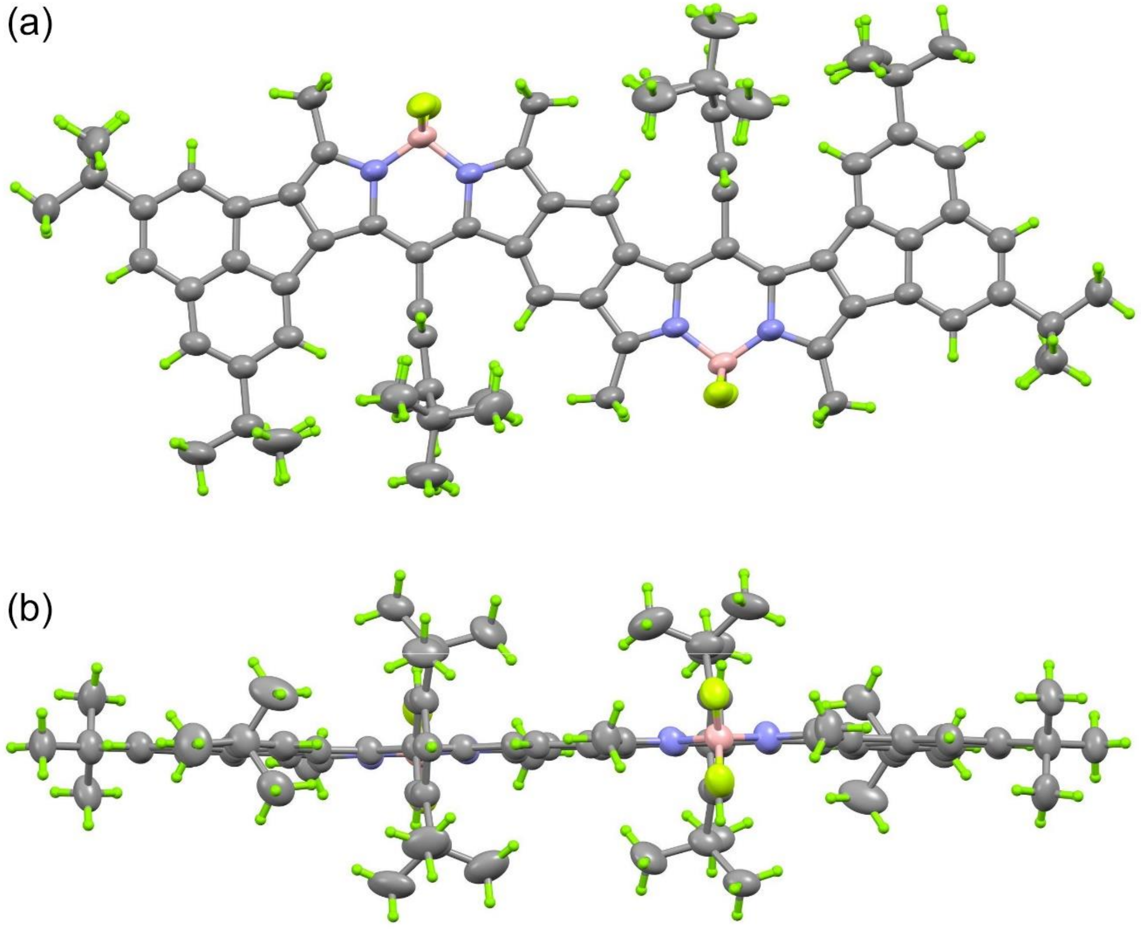

Figure S6. Ortep drawing of **10b** (top (a) and side (b) views). The structure without solvent molecules (CH_2Cl_2 and acetoniteile) was refined by the Platon Squeeze technique. Disordered substituents with less occupancy are omitted for clarity.

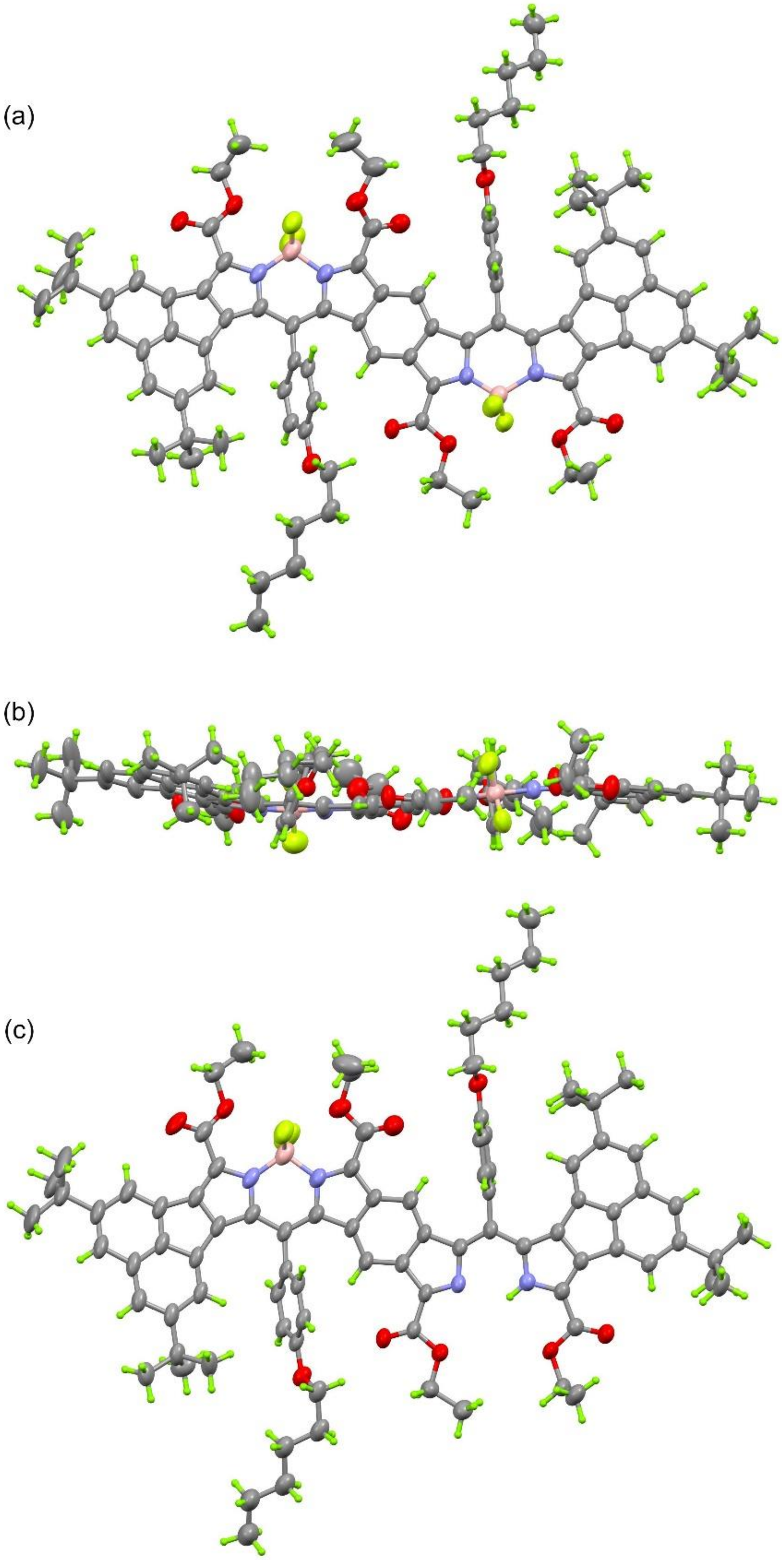

Figure S7. Ortep drawing of top (a) and side (b) views of **11c** and top view (c) of **17**. Disordered substituents with less occupancies are omitted for clarity.

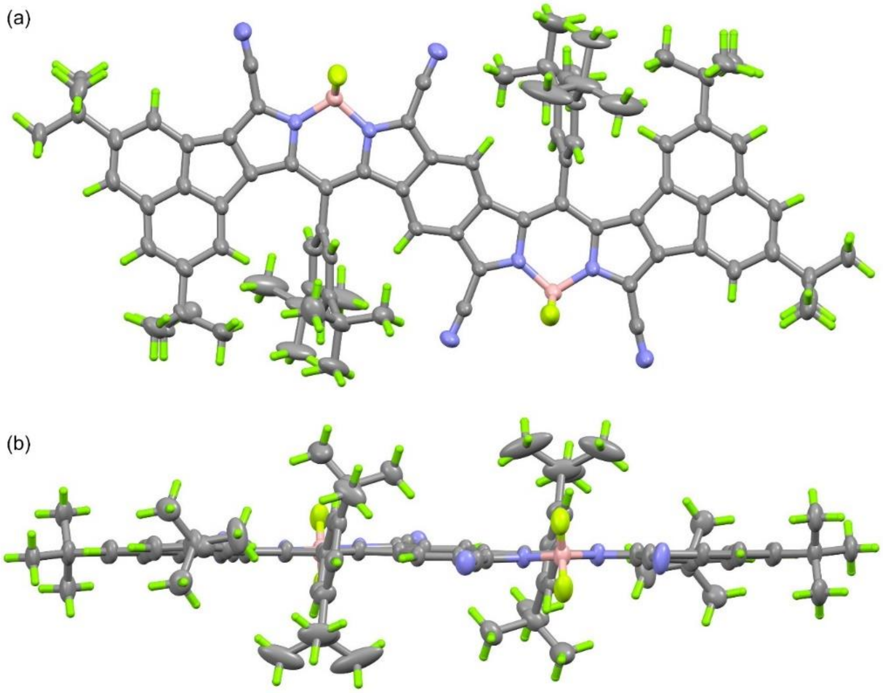

Figure S8. Ortep drawing of **12b** (top (a) and side (b) views). Solvent molecules and disordered substituents with less occupancy are omitted for clarity.

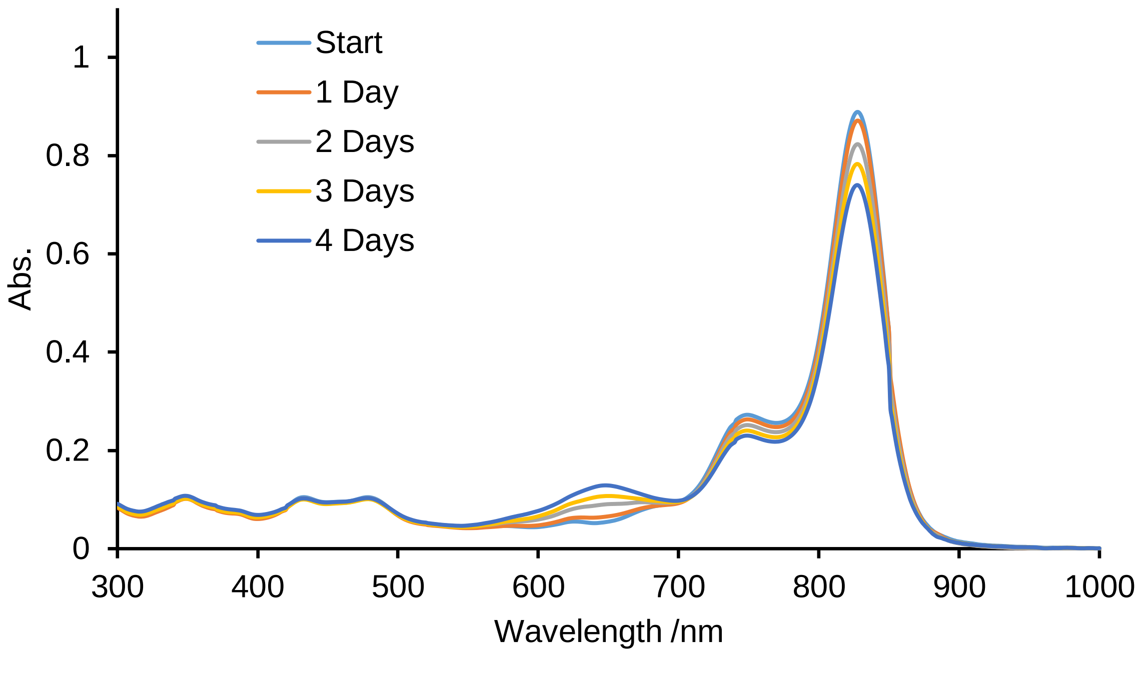

Figure S9. UV-vis-NIR monitoring of **10a** in spectroscopic-grade CH_2Cl_2

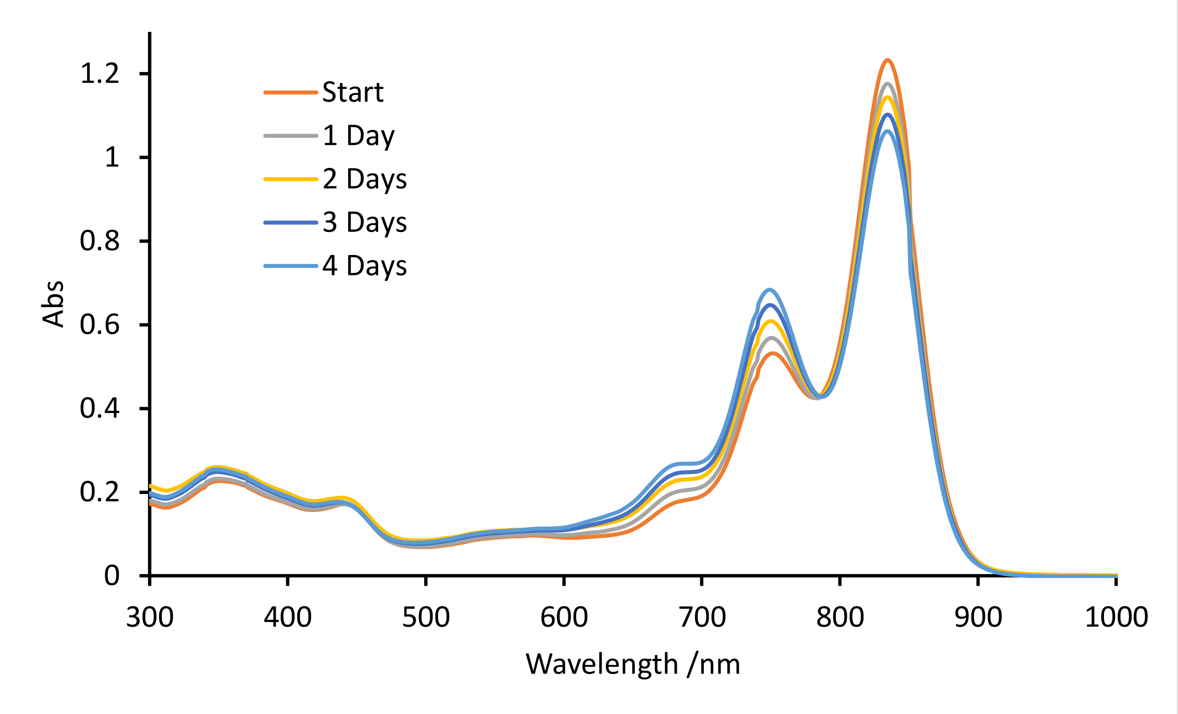

Figure S10. UV-vis-NIR monitoring of **11b** in spectroscopic-grade CH_2Cl_2

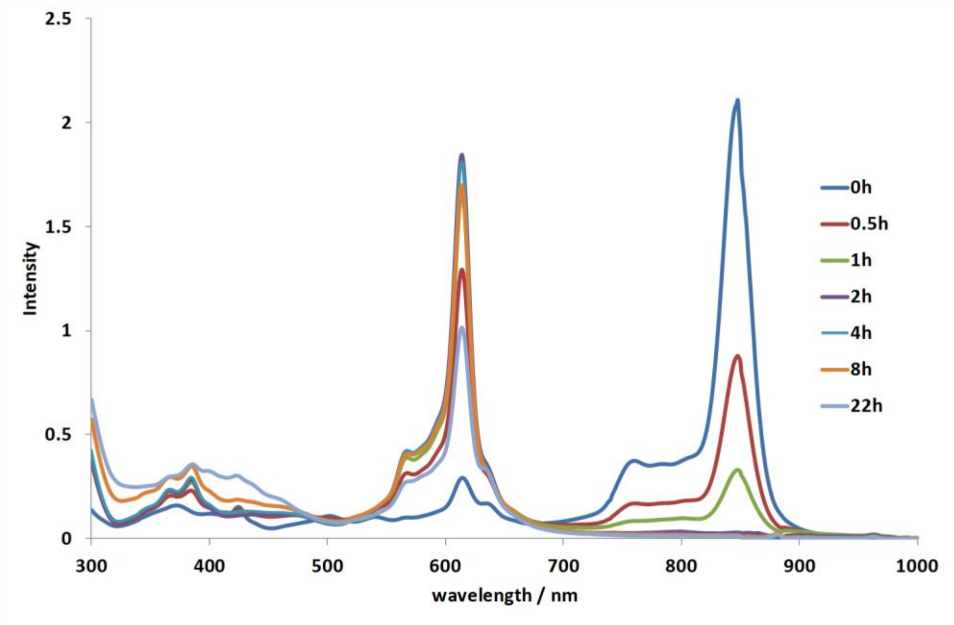

Figure S11. UV-vis-NIR monitoring of **1a** (CH_2Cl_2) under air in a room light.

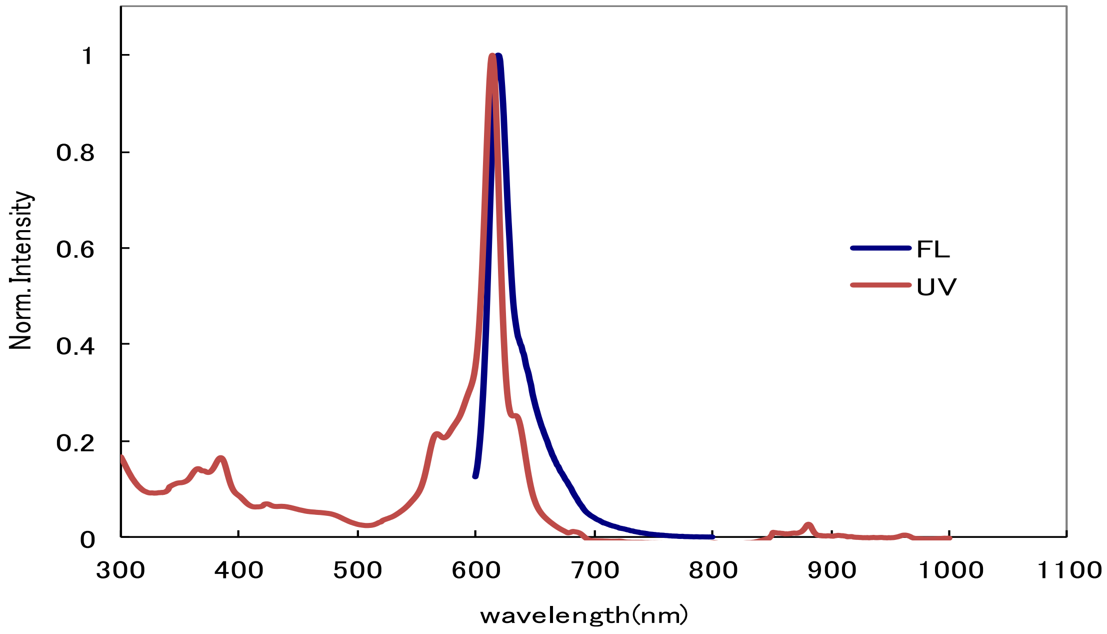

Figure S12. UV-vis-NIR and fluorescence spectra of decomposed **1a** in CH_2Cl_2

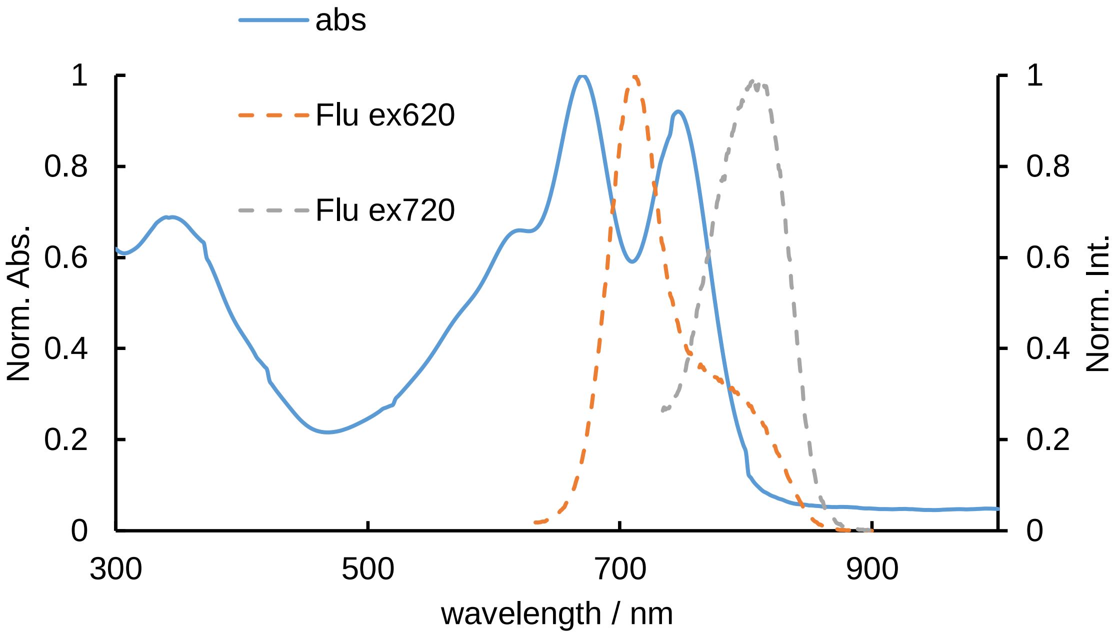

Figure S13. UV-vis-NIR and fluorescence spectra of decomposed **11c** after *ca*. 30 days.

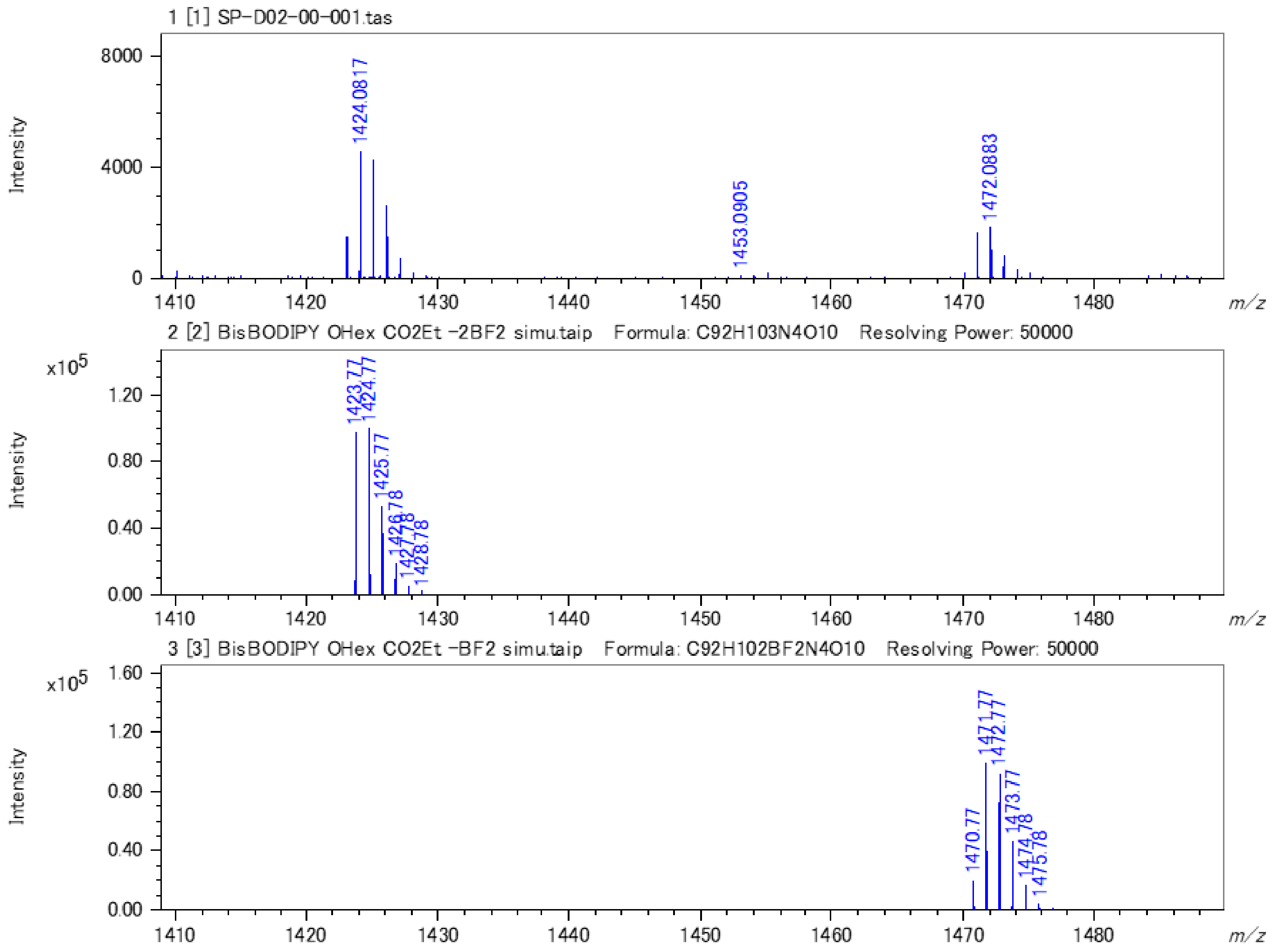

Figure S14. MALDI-TOF MS spectrum of the decomposed sample of **11c** (upper case) and simulated spectra of **18** (middle case) and **17** (lower case)

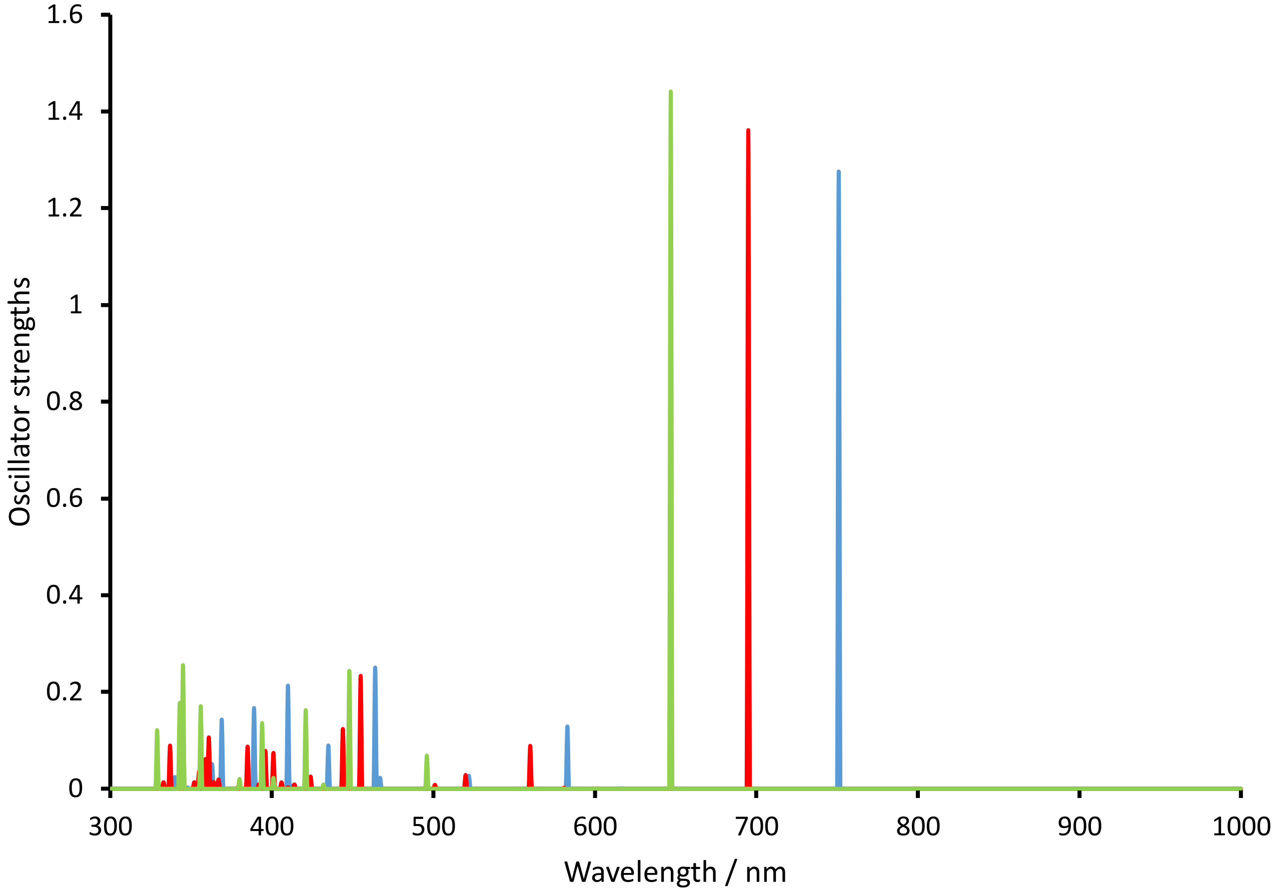

Figure S15. TD-DFT calculation spectra of **15** (blue) and the corresponding mono-(red) and di-decomplexed (green) compounds

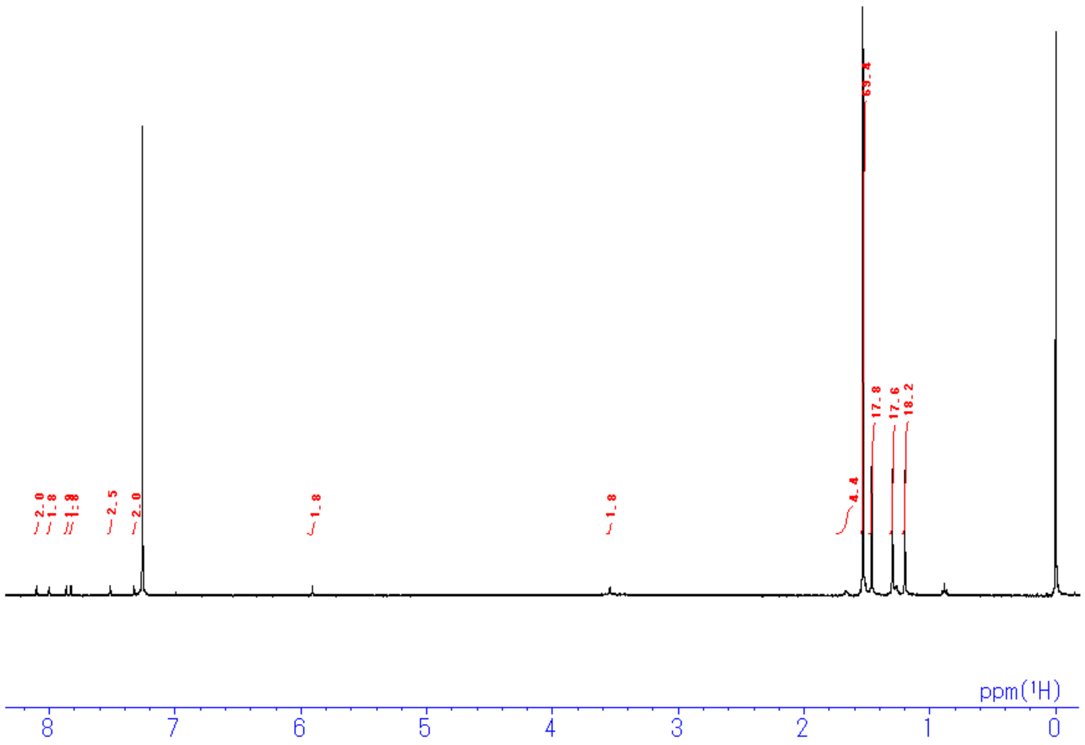

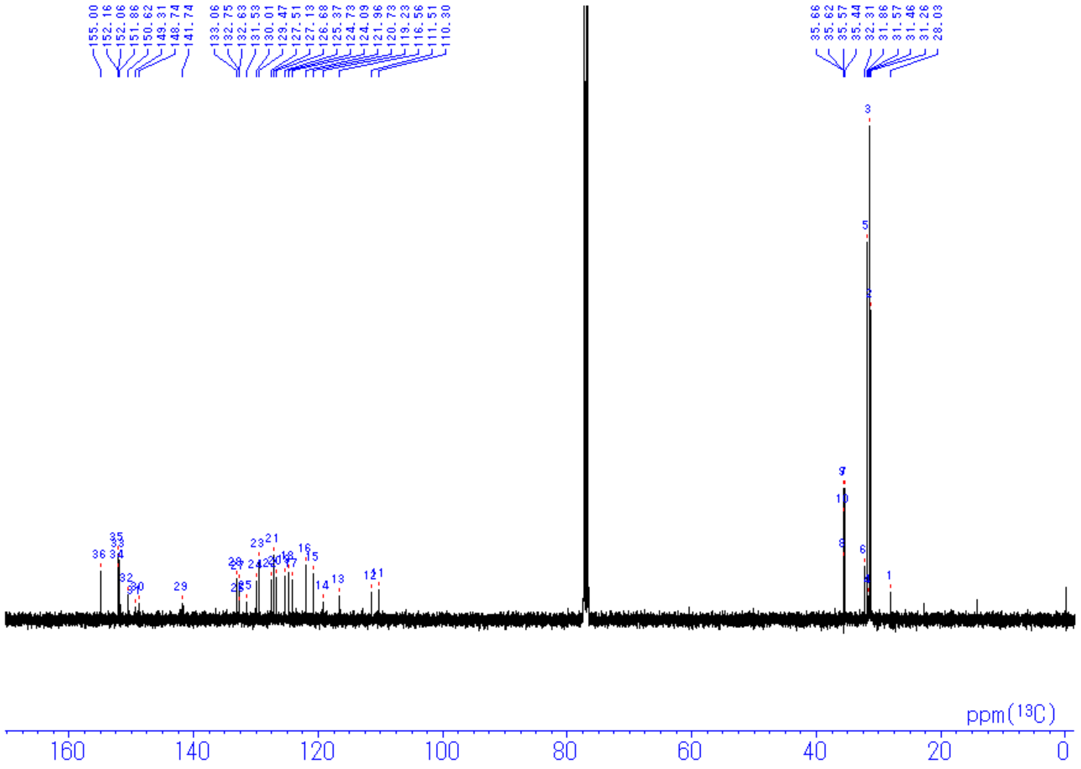

Figure S16. ^{1}H (upper) and ^{13}C (lower) NMR spectra of **9b**

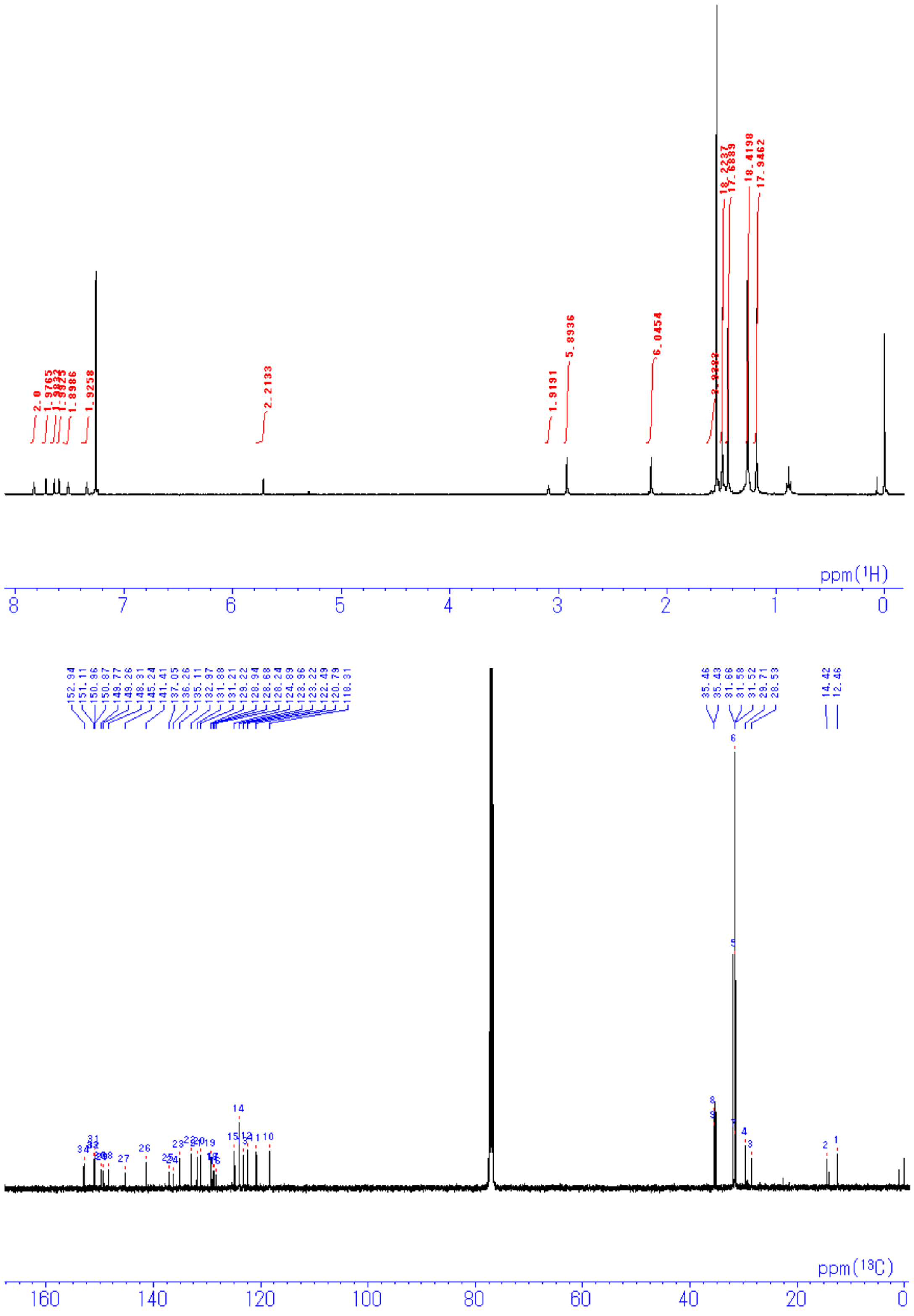

Figure S17. ^{1}H (upper) and ^{13}C (lower) NMR spectra of **7b**

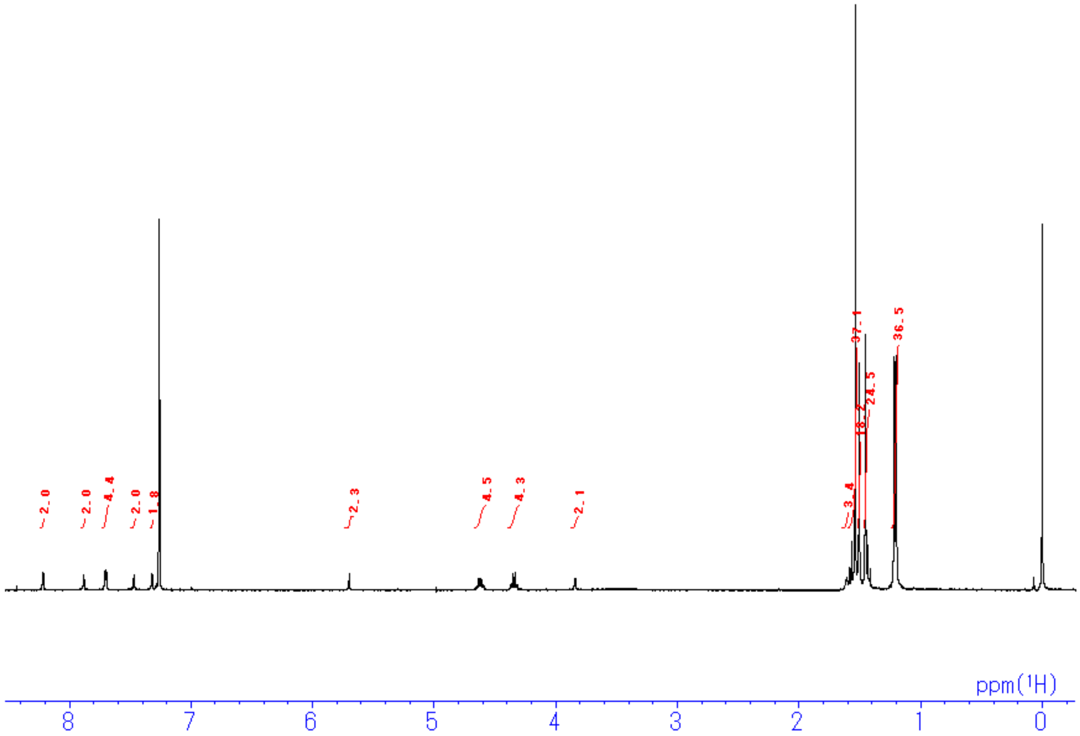

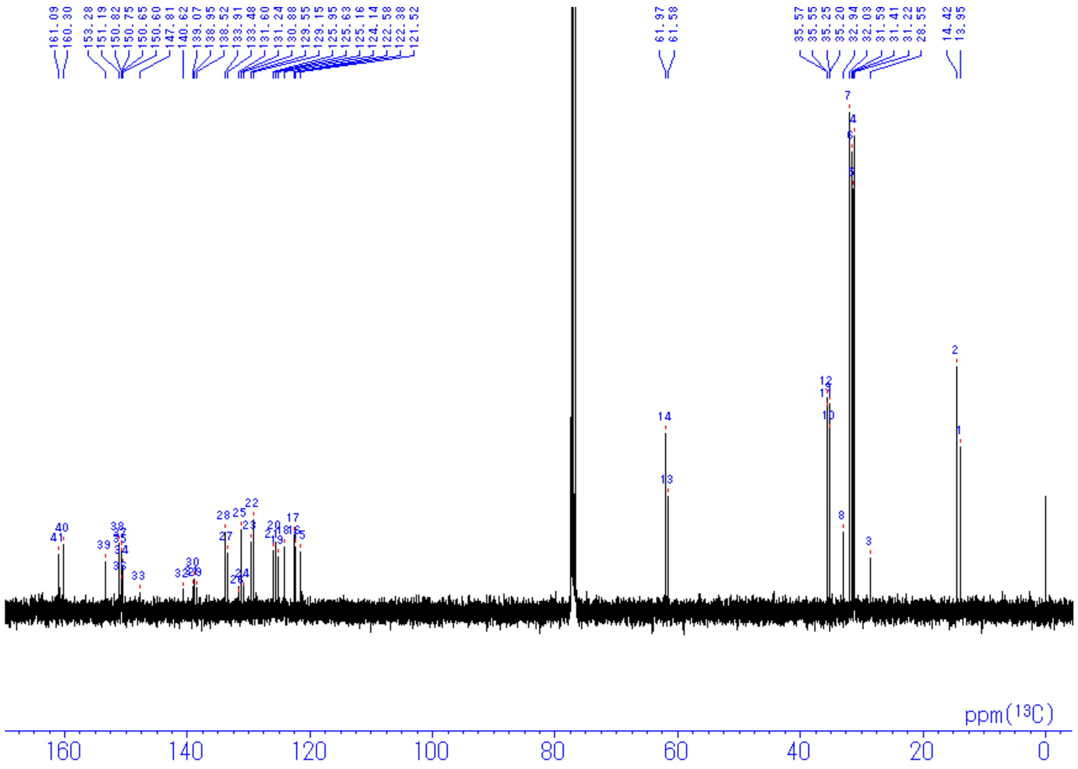

Figure S18. ^{1}H (upper) and ^{13}C (lower) NMR spectra of **8b**

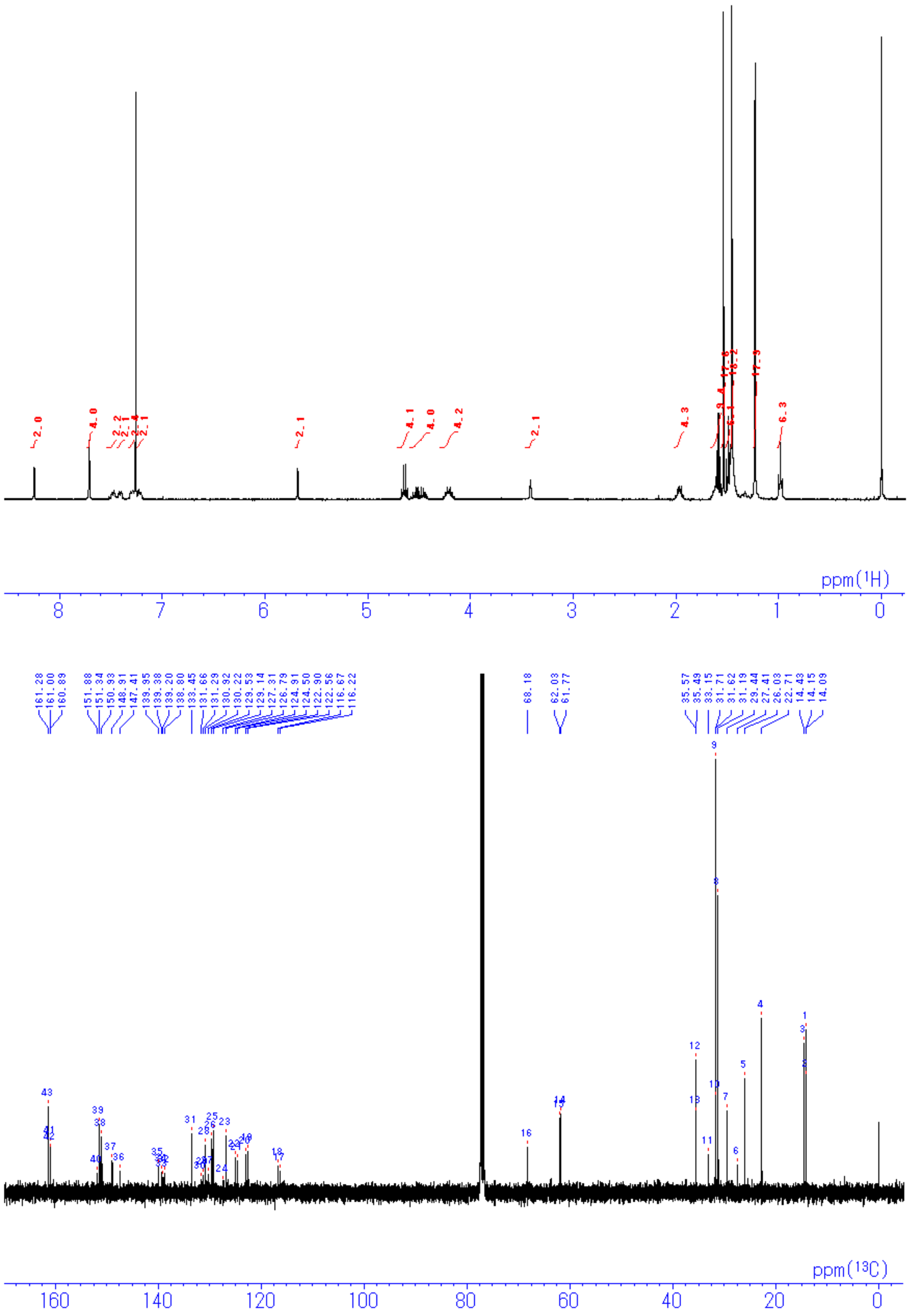

Figure S19. ^{1}H (upper) and ^{13}C (lower) NMR spectra of **8c**

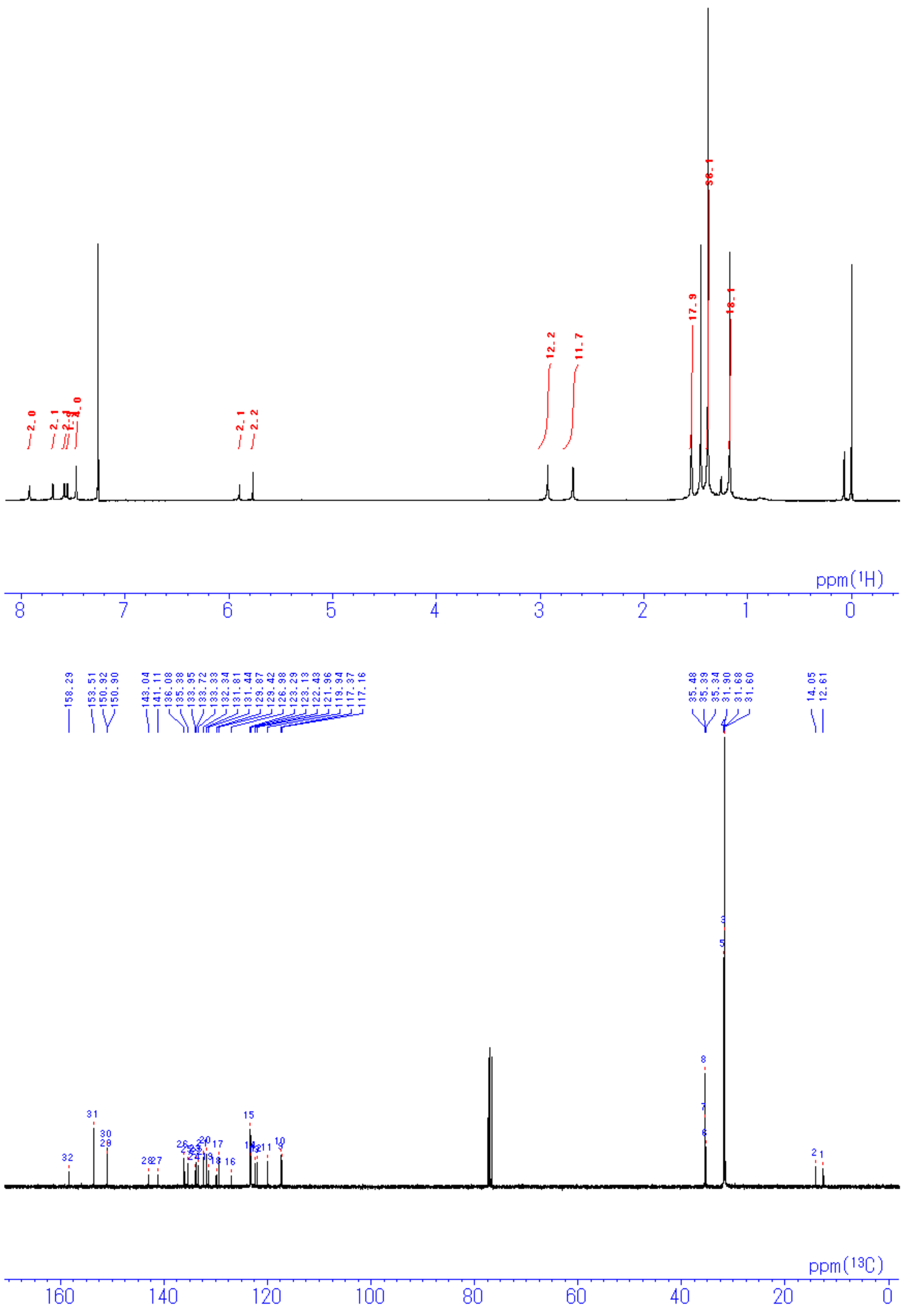

Figure S20. ^{1}H (upper) and ^{13}C (lower) NMR spectra of **10b**

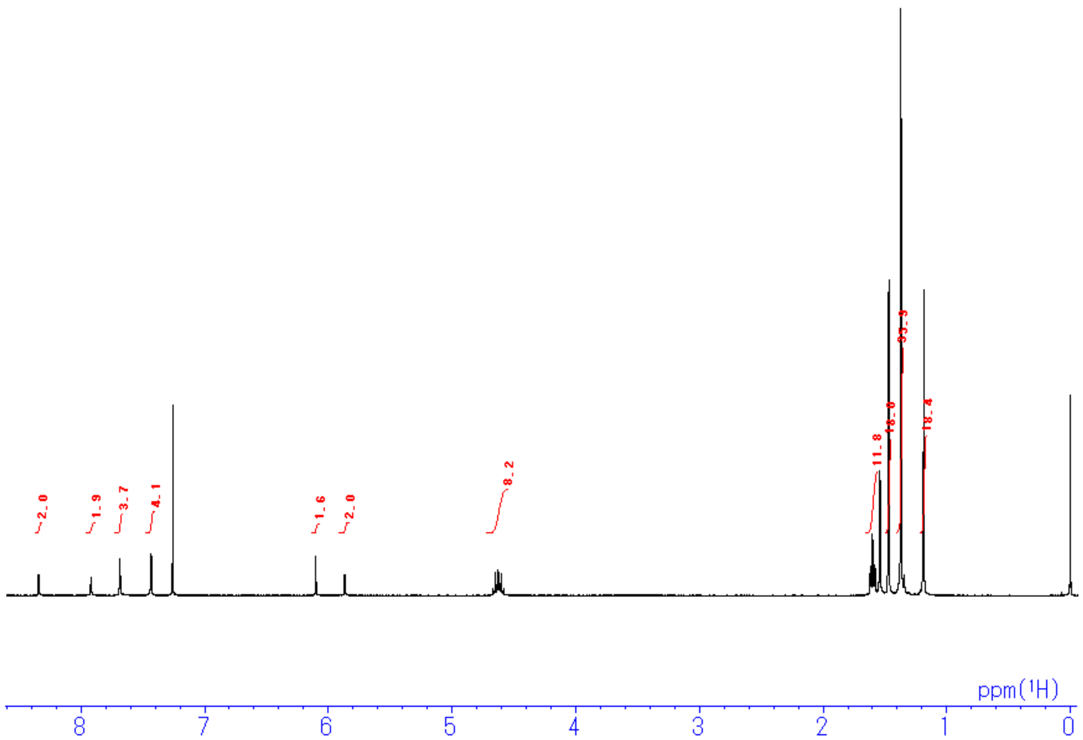

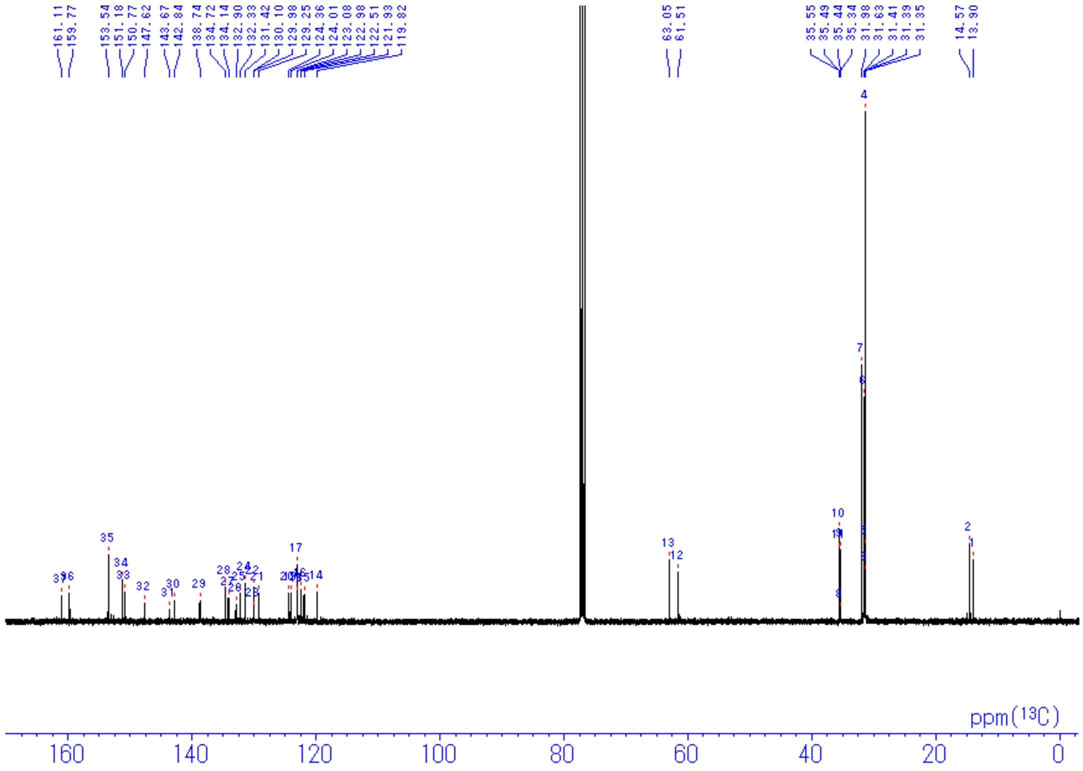

Figure S21. ^{1}H (upper) and ^{13}C (lower) NMR spectra of **11b**

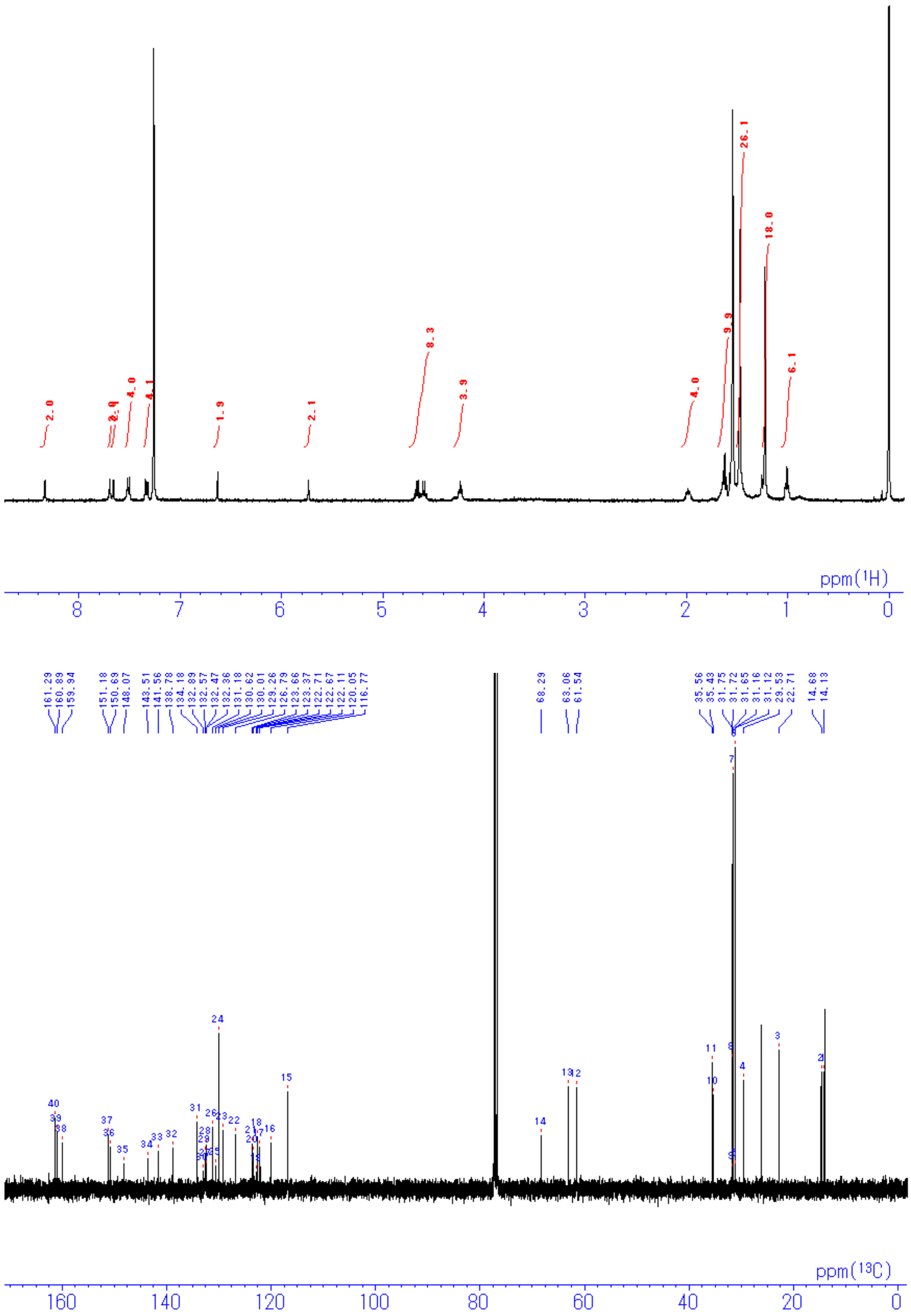

Figure S22. ^{1}H (upper) and ^{13}C (lower) NMR spectra of **11c**

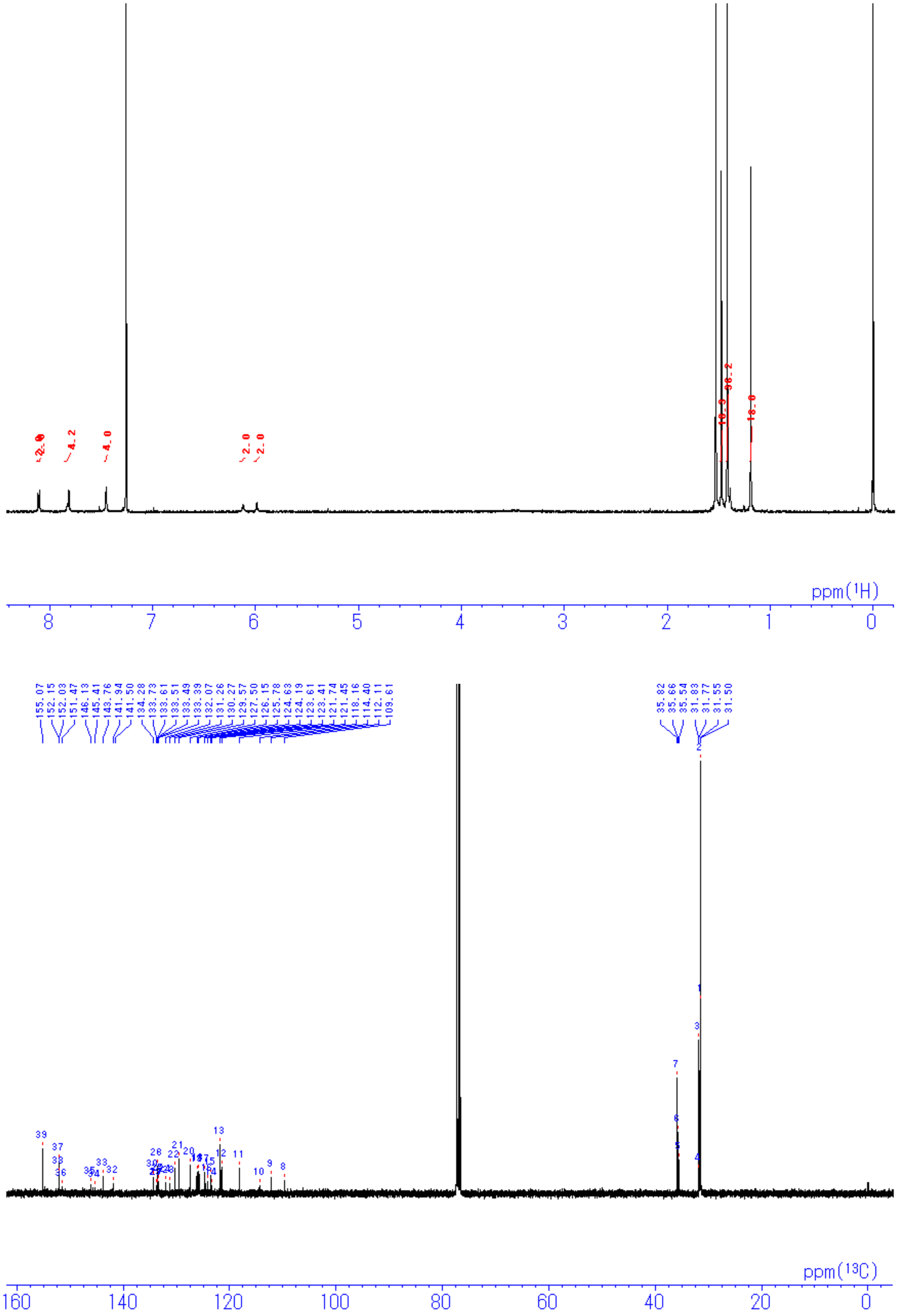

Figure S23. ^{1}H (upper) and ^{13}C (lower) NMR spectra of **12b**
